# A highly accurate Hermite polynomial-based least-squares approach for solving fractional Volterra-Fredholm integro-differential equations

**DOI:** 10.1371/journal.pone.0346080

**Published:** 2026-04-07

**Authors:** Maha M. Hamood, Abdulrahman A. Sharif, Kirtiwant P. Ghadle

**Affiliations:** 1 Department of Mathematics, Taiz University, Taiz, Yemen; 2 Department of Mathematics, Hodeidah University, AL-Hudaydah, Yemen; 3 Department of Mathematics, Dr. Babasaheb Ambedkar Marathwada University, Aurangabad, Maharashtra, India; University of Education, PAKISTAN

## Abstract

This paper presents a comprehensive numerical study on the efficacy of a Hermite polynomial-based least-squares method for solving Volterra–Fredholm fractional integro-differential equations (V-FFIDEs). In our approach, we construct an approximate solution as a finite expansion of Hermite polynomials. This trial solution is systematically substituted into the governing V-FFIDE. Following the analytical evaluation of the fractional and integral operators, we formulate a residual function. The core of our method involves minimizing the squared norm of this residual over the problem domain, a process that transforms the original problem into a well-defined system of linear algebraic equations. To validate our methodology, we conducted a series of numerical experiments on a collection of representative examples. The results of our study, presented through detailed tables of numerical outcomes and comparative graphical illustrations, conclusively demonstrate the high accuracy, computational efficiency, and robust convergence of the proposed technique.

## 1 Introduction

Fractional calculus, which generalizes classical calculus to derivatives and integrals of arbitrary real or complex order, has transitioned from mathematical abstraction to an essential modeling framework across engineering and scientific disciplines. While its foundations trace back to Leibniz, Riemann, and Liouville, contemporary applications of fractional differential equations (FDEs) and fractional integro-differential equations (FIDEs) have demonstrated exceptional capability in capturing phenomena characterized by memory effects, hereditary properties, and anomalous diffusion [1–4].

The practical significance of fractional Volterra-Fredholm integro-differential equations extends across numerous scientific and engineering domains where memory effects and non-local interactions are paramount. In viscoelastic material science, these equations model the complex stress-strain relationships in polymers, biological tissues, and asphalt, where the fractional derivative captures the power-law relaxation behavior characteristic of such materials [1]. The Volterra integral component, with its variable upper limit *b*(*s*), naturally represents the accumulation of historical deformation (the material’s memory), while the Fredholm term accounts for long-range spatial interactions throughout the material volume. In transport phenomena, VFIDEs are pivotal for describing anomalous diffusion processes observed in highly heterogeneous porous media, such as contaminant transport in groundwater aquifers or nutrient diffusion in fractals like lungs and river networks [4]. Here, the fractional order is linked to the heterogeneity of the medium, and the Fredholm operator can model diffusion through a complex, fixed structure. Biological systems provide another rich application domain. These equations can model neuronal dynamics with memory effects, where the Volterra term represents the accumulation of past electrical activity and the Fredholm component captures spatial interactions along the axon. Similarly, in epidemiology, VFIDEs can describe disease spread in a spatially structured population, where infection rates depend on both recent contact history (Volterra) and global population mixing (Fredholm).

The enhanced modeling capability of fractional operators, however, introduces significant computational challenges. The non-local character of fractional derivatives renders analytical solutions for most FIDEs intractable, creating a pressing need for robust and efficient numerical schemes. This challenge has motivated extensive research into numerical methods, with spectral techniques employing orthogonal polynomials emerging as particularly promising approaches. These methods offer the potential for high-order accuracy and exponential convergence rates for sufficiently smooth solutions [5], making them attractive for fractional problems.

The literature reveals diverse methodological approaches to fractional integro-differential equations. Researchers have explored standard least-squares methods with constructed orthogonal polynomials for linear FIDEs [6–8], specialized treatments for Volterra-type equations [9,10], perturbed variational iteration methods [11], and polynomial-based discretizations using Bernstein polynomials [12]. Additional investigations include custom orthogonal polynomials [13], Hermite polynomial approximations [14,15], and collocation schemes with polynomial splines [16]. Recent advances continue to expand this methodological landscape, including improved block-pulse functions for mixed Volterra-Fredholm integral equations [17], novel Bernoulli polynomial approaches for Fredholm equations [18], and hybrid techniques combining integral transforms with Hermite polynomials for fractional Fredholm-Volterra problems [19]. A common theme across these studies is the pursuit of high accuracy and robust convergence through orthogonal function expansions.

Among recent spectral approaches, notable contributions include a new spectral framework for solving fractional-order differential equations that achieves improved efficiency without requiring residual functions [20]. This approach demonstrates the ongoing optimization of spectral methods for fractional problems. Additionally, high-precision spectral methods have been developed specifically for Bagley–Torvik equations [21], showcasing the effectiveness of spectral techniques for specialized fractional differential equations. For boundary value problems in fractional calculus, advanced numerical techniques have been successfully applied to obtain accurate solutions [22], further validating the robustness of spectral approaches in fractional settings.

Despite these advances, significant gaps remain in the current literature. Most existing works address either Volterra-type or Fredholm-type fractional integro-differential equations separately [10], or employ polynomial bases that may be suboptimal for problems on unbounded or semi-infinite domains. While recent hybrid methods show promise [19], they often combine multiple mathematical frameworks, which can complicate implementation and theoretical analysis. The potential of a unified Hermite polynomial-based least-squares framework specifically designed for general Volterra-Fredholm fractional integro-differential equations (VFIDEs) remains largely unexplored. Hermite polynomials, with their natural orthogonality under Gaussian weight and excellent approximation properties, offer a promising basis for problems with oscillatory or rapidly varying solutions.

The numerical method developed in this work is particularly suited for real-world applications because it efficiently handles the combined challenges of fractional derivatives (capturing memory effects), Volterra integrals (modeling cumulative historical influences), and Fredholm integrals (representing global spatial interactions). The Hermite polynomial basis offers advantages for problems with solutions exhibiting rapid variations or oscillatory behavior, common in many physical applications.

The choice of Hermite polynomials over other orthogonal bases (e.g., Legendre or Chebyshev) is motivated by their natural suitability for functions exhibiting rapid decay or oscillatory behavior, which are common in fractional dynamical systems. Even when mapped to the finite interval [0,1], Hermite expansions retain strong approximation properties and can achieve spectral convergence for smooth solutions, making them an efficient basis for the proposed global least-squares framework.

This study aims to address these limitations through a two-pronged approach:

- To develop a comprehensive Hermite polynomial-based least-squares approximation method for numerical solution of general linear VFIDEs of fractional order, and
- To rigorously assess the method’s accuracy, stability, and convergence properties through systematic numerical experiments with known exact solutions.

The primary contribution of this work lies in bridging the gap between least-squares spectral approximations and Hermite polynomial bases for fractional integro-differential problems encompassing both Volterra and Fredholm integral components. This unified framework enhances computational efficiency while maintaining high accuracy, thereby extending the applicability of least-squares spectral methods to a broader class of fractional problems [5,23,24].

Building upon the foundational work in [25], which explored Least-Squares Approximation Methods for Volterra fractional integro-differential equations using Hermite polynomials, and inspired by recent advances in hybrid methods [19], the current research generalizes this framework to handle the more complex case of VFIDEs with both variable-limit Volterra and fixed-limit Fredholm integral components.

This paper focuses on obtaining numerical solutions for a general ${𝔴}^{\text{th}}$-order linear Volterra-Fredholm integro-differential equation (VFIDE) of the form:


$${𝔇}^{𝔴}𝕜(𝔰)=\rho (𝔰)+𝔮(𝔰)f(𝔰)+\int_{a}^{b(𝔰)}𝕜(𝔰,𝔭)𝕜(𝔭)d𝔭+\int_{a}^{T}𝒢(𝔰,𝔭)𝕜(𝔭)d𝔭,\;𝔰\in [a,T]$$ (1)

subject to the initial conditions:


$${𝕜}^{\left(j\right)}(\zeta )=\zeta_{i};\;j=0,1,\cdots ,\upsilon -1$$ (2)

where:

- υ=⌈𝔴⌉ is the smallest integer greater than or equal to 𝔴,
- ${𝔇}^{𝔴}$ denotes the Caputo fractional derivative of order 𝔴>0,
- ρ(𝔰), 𝔮(𝔰), and f(𝔰) are known continuous source functions on the interval [*a*, *T*],
- 𝕜(𝔰,𝔭) is the kernel of the Volterra integral part, with a variable upper limit b(𝔰), where a≤b(𝔰)≤T,
- 𝒢(𝔰,𝔭) is the kernel of the Fredholm integral part, defined on the fixed domain [a,T]×[a,T],
- ζ is a point in [*a*, *T*] where the initial conditions are specified, and $\zeta_{i}$ are given real constants.

Definition and Regularity of the Kernels. In the general form of the problem (1), the functions 𝕜(𝔰,𝔭) and 𝒢(𝔰,𝔭) are biberred to as the kernel functions of the Volterra and Fredholm integrals, respectively. The kernel 𝕜(𝔰,𝔭) determines the local (history-dependent) interaction between the current variable 𝔰 and the past variable 𝔭 within the interval [a,b(𝔰)], whereas 𝒢(𝔰,𝔭) describes the global coupling over the fixed domain [*a*,*T*]. Throughout this work, both kernels are assumed to be continuous or to possess at most *integrable (weak) singularities* with respect to 𝔭, such that


$$\int_{a}^{b(𝔰)}|𝕜(𝔰,𝔭)|\;d𝔭<\infty ,\;\int_{a}^{T}|𝒢(𝔰,𝔭)|\;d𝔭<\infty ,\;\forall \;𝔰\in [a,T].$$

These conditions ensure the existence of the integrals and the applicability of the proposed collocation scheme. Typical examples of admissible kernels include smooth functions such as 𝕜(𝔰,𝔭)=(𝔰−𝔭), $𝕜(𝔰,𝔭)={𝔰}^{2}𝔭$, or $𝕜(𝔰,𝔭)=\frac{e^{-𝔰𝔭}}{1+\sqrt{𝔭}}$, and weakly singular forms such as $(𝔰-𝔭{)}^{-\alpha }$ with 0≤α<1. The analysis and numerical scheme developed in this work are guaranteed to be applicable under these conditions. Kernels exhibiting non-integrable (strong) singularities fall outside this framework and would require specialized treatment, which is beyond the scope of this study.

The primary objective of this study is to construct a stable and convergent numerical solution ${𝕜}_{𝒩}(𝔰)$ to the problem (1)-(2) using a least squares approximation scheme with Hermite polynomials as basis functions. The specific goals include:

1. To develop a computational algorithm for transforming the generalized VFIDE into a system of linear algebraic equations.
2. To provide a rigorous analysis of the convergence properties and stability of the proposed method under reasonable assumptions on the kernels and source terms.
3. To demonstrate the efficacy and accuracy of the method through numerical experiments on test problems with known solutions.
4. To investigate the method’s performance for different forms of the variable limit b(𝔰) and to compare its efficiency with existing numerical techniques.

The remaining content is structured as follows: Section 2 deals with the fundamental ideas, definitions, and theorems that will underpin the results. In Sec. 3, the proposed Hermite polynomial-based least-squares methodology is described in detail. Section 4 is devoted to the formulation and Analysis of the Proposed Method. Finally, in Section 5, numerical experiments and results are presented and discussed.

## 2 Preliminaries

This section presents the fundamental mathematical concepts, function spaces, and tools that form the basis of the proposed numerical method.

**Definition 1** [1]*. Let* 𝔴>0 *with* n−1<𝔴<n*,* n∈ℕ*, and let* $𝕜\in AC^{n}[a,T]$*, the space of functions with absolutely continuous n-th derivative. The Caputo fractional derivative of order* 𝔴 *is defined as*


$${𝔇}_{C}^{𝔴}𝕜(𝔰)=\frac{1}{\Gamma (n-𝔴)}\int_{a}^{𝔰}(𝔰-𝔭{)}^{n-𝔴-1}{𝕜}^{\left(n\right)}(𝔭)\;d𝔭.$$

**Definition 2** [26] *For* 0<𝔴<1*, the fractional Sobolev space* $H^{𝔴}(0,1)$ *is defined as*


$$H^{𝔴}(0,1)=\left\{𝕜\in L^{2}(0,1):\int_{0}^{1}\int_{0}^{1}\frac{|𝕜(𝔰)-𝕜(𝔭){|}^{2}}{|𝔰-𝔭{|}^{1+2𝔴}}d𝔰d𝔭<\infty \right\},$$

*endowed with the norm* $\|𝕜{\|}_{H^{𝔴}(0,1)}^{2}=\|𝕜{\|}_{L^{2}(0,1)}^{2}+|𝕜{|}_{H^{𝔴}(0,1)}^{2}$.

**Definition 3** [5,23]*. Two functions* $z_{n}(𝔰)$ *and* $z_{m}(𝔰)$ *are said to be orthogonal on the interval [a, b] with respect to a weight function* w(𝔰)>0 *if:*


$$\langle z_{n}(𝔰),z_{m}(𝔰)\rangle =\int_{a}^{b}w(𝔰)z_{n}(𝔰)z_{m}(𝔰)d𝔰=0,\;n\neq m.$$ (3)

**Definition 4** [24,27]*. Hermite polynomials, denoted as* $H_{n}(𝔰)$*, are a classical set of orthogonal polynomials on* (−∞,∞) *with respect to the weight function* $w(𝔰)=e^{-{𝔰}^{2}}$*. They satisfy:*


$$\int_{-\infty }^{\infty }e^{-{𝔰}^{2}}H_{n}(𝔰)H_{m}(𝔰)d𝔰=2^{n}n!\sqrt{\pi }\delta_{nm}.$$

*They can be generated by the recurrence relation:*


$$\begin{array}{cc} H_{0}(𝔰) & =1,\\ H_{1}(𝔰) & =2𝔰,\\ H_{n+1}(𝔰) & =2𝔰H_{n}(𝔰)-2nH_{n-1}(𝔰)\;\text{for }n\geq 1. \end{array}$$

### 2.1 Normalization to the unit interval

[5,23]. As the physical domain of the problem is [*a*, *T*], we map Hermite polynomials to the unit interval [0, 1] via the linear transformation 𝔭=(𝔰−a)/(T−a). For [0, 1], this yields 𝔭=𝔰. The basis functions are $H_{n}(𝔰)$ for 𝔰∈[0,1], denoted ${\tilde{H}}_{n}(𝔰)$. This mapping sacrifices orthogonality but preserves linear independence, and the set $\left\{{\tilde{H}}_{n}(𝔰)\right\}$ remains a complete basis for *L*^2^(0,1) [23,28].

### 2.2 Residual error analysis

Following established practice in numerical analysis [29], we complement our solution error analysis with *residual error* measurements. The residual provides a fundamental assessment of how well the numerical solution satisfies the original governing equation.: We consider the nonlinear fractional Volterra–Fredholm integro-differential equation given in Problem (1). For clarity, the problem is rewritten in operator form as


ℒ[𝕜](s)=ρ(s),s∈[a,T], (4)

where the nonlinear operator ℒ:X→X is defined by


$$\begin{array}{cc} ℒ[𝕜](s) & ={𝔇}^{w}𝕜(s)-𝔮(s)f(s)\\ & \;-\int_{a}^{b(s)}𝕜(s,p)𝕜(p)\;dp-\int_{a}^{T}𝒢(s,p)𝕜(p)\;dp. \end{array}$$

Here, *X* denotes a suitable Banach space such as *C*[*a*,*T*] or *L*^2^[*a*,*T*].

Let 𝕜∈X be the exact solution of (4), and let ${𝕜}_{N}\in X_{N}\subset X$ be its numerical approximation obtained using the proposed method with *N* basis functions.

#### 2.2.1 Residual definition.

The residual associated with the numerical solution ${𝕜}_{N}$ is defined as


$$ℛ[{𝕜}_{N}](s):=ℒ[{𝕜}_{N}](s)-\rho (s),\;s\in [a,T].$$ (5)

By definition of the exact solution, ℛ[𝕜](s)=0 for all s∈[a,T]. Hence, $ℛ[{𝕜}_{N}]$ measures the deviation of the numerical approximation from satisfying the original governing equation.

#### 2.2.2 Residual error norms.

To quantify the residual error, we introduce the following norms:


$$\|ℛ{\|}_{L^{\infty }}=\max_{s\in [a,T]}\left|ℛ[{𝕜}_{N}](s)\right|,$$ (6)

$$\|ℛ{\|}_{L^{2}}={\left(\int_{a}^{T}{\left|ℛ[{𝕜}_{N}](s)\right|}^{2}ds\right)}^{1/2}.$$ (7)

The *L*^∞^-norm captures the maximum pointwise defect, while the *L*^2^-norm measures the global mean-square consistency of the numerical approximation.

#### 2.2.3 Analytical assumptions.

We assume the following standard conditions:

(A1) The fractional derivative operator ${𝔇}^{w}$ is linear and bounded on *X*.
(A2) The kernel functions 𝕜(s,p) and 𝒢(s,p) are continuous and bounded.
(A3) The operator ℒ satisfies a Lipschitz condition:

‖ℒ[u]−ℒ[v]‖≤L‖u−v‖,∀u,v∈X,

for some constant *L* > 0.

**Lemma 1 (Residual Identity).** *Let* 𝕜 *be the exact solution of* (4) *and* ${𝕜}_{N}$ *its numerical approximation. Then*


$$ℒ[{𝕜}_{N}]-ℒ[𝕜]=ℛ[{𝕜}_{N}].$$

**Proof 1** *Since* ℒ[𝕜]=ρ *by definition of the exact solution and* $ℛ[{𝕜}_{N}]=ℒ[{𝕜}_{N}]-\rho$*, we obtain*


$$ℒ[{𝕜}_{N}]-ℒ[𝕜]=ℒ[{𝕜}_{N}]-\rho =ℛ[{𝕜}_{N}],$$

*which completes the proof.*

**Theorem 1 (Residual-Based Error Estimate).** *Under assumptions (A1)–(A3), there exists a constant C > 0, independent of N, such that*


$$\|𝕜-{𝕜}_{N}\|\leq C\;\|ℛ[{𝕜}_{N}]\|.$$ (8)

**Proof 2** *By Lemma 1 and the Lipschitz continuity of* ℒ*, we have*


$$\|ℛ[{𝕜}_{N}]\|=\|ℒ[{𝕜}_{N}]-ℒ[𝕜]\|\leq L\|{𝕜}_{N}-𝕜\|.$$

*If* ℒ *is invertible and its inverse is bounded, then*


$$\|{𝕜}_{N}-𝕜\|\leq \|{ℒ}^{-1}\|\;\|ℛ[{𝕜}_{N}]\|.$$

*Setting* $C=\|{ℒ}^{-1}\|$ *yields the desired estimate.*

**Corollary 1 (Convergence)** *If*


$$\lim_{N\to \infty }\|ℛ[{𝕜}_{N}]\|=0,$$

*then*


$$\lim_{N\to \infty }\|𝕜-{𝕜}_{N}\|=0.$$

#### 2.2.4 Discussion.

The above analysis establishes that the residual error provides a rigorous and reliable indicator of numerical accuracy. In particular, for nonlinear fractional Volterra–Fredholm integro-differential equations where exact solutions are generally unavailable, residual convergence guarantees consistency, stability, and convergence of the proposed numerical method.

### 2.3 Relationship between residual and true error

The residual error and true error are two distinct but related measures of numerical accuracy.

- Residual Error: The residual $ℛ[{𝐤}_{N}](s)$ measures how well the approximate solution **k**_*N*_(*s*) satisfies the governing equation (4). It is defined as
  $$ℛ[{𝐤}_{N}](s)=ℒ[{𝐤}_{N}](s)-\rho (s).$$
  A small residual indicates that the numerical solution closely satisfies the original equation pointwise.
- True Error: The true error *E*(*s*) measures the deviation of the approximate solution from the exact (analytical) solution **k**(*s*):
  $$E(s)={𝐤}_{N}(s)-𝐤(s).$$
  This metric is only computable when the exact solution is known.
- Relationship: Under the assumptions (A1)–(A3) and if the operator ℒ is invertible with bounded inverse, the residual provides an upper bound for the true error (Theorem 1):
  $$\|𝐤-{𝐤}_{N}\|\leq C\|ℛ[{𝐤}_{N}]\|.$$
  Thus, a decreasing residual norm implies a decreasing true error norm, ensuring convergence of the method.
- Role in Accuracy Assessment:
  1. The residual is a *reliability indicator*—it assesses the consistency of the numerical solution with the governing equation, even when the exact solution is unknown.
  2. The true error is a *validation metric*—it quantifies the actual accuracy of the approximation when an exact solution is available.
  3. In practice, the residual is often used to monitor convergence and stability, while the true error is used to verify and benchmark the method.

In the numerical experiments (Section 5), we report both residuals and true errors where exact solutions are known. The consistent decay of both metrics with increasing *N* confirms the robustness and spectral accuracy of the proposed Hermite least-squares method.

## 3 Methodology

### 3.1 Mathematical formulation

Having defined the Hermite polynomial basis in Section 2, we now describe the least-squares algorithm for solving the generalized fractional Volterra–Fredholm integro-differential equation (VFIDE):


$${𝔇}^{𝔴}𝕜(𝔰)=\rho (𝔰)+𝔮(𝔰)f(𝔰)+\int_{a}^{b(𝔰)}𝕜(𝔰,𝔭)𝕜(𝔭)\;d𝔭+\int_{a}^{T}𝒢(𝔰,𝔭)𝕜(𝔭)\;d𝔭,\;𝔰\in [a,T],$$ (9)

subject to the initial conditions:


$${𝕜}^{\left(j\right)}(a)=\zeta_{j},\;j=0,1,\ldots ,\upsilon -1,\;\upsilon =\lceil 𝔴\rceil .$$ (10)

We seek an approximate solution ${𝕜}_{𝒩}(𝔰)$ in the finite-dimensional subspace ${𝒮}_{𝒩}=\mathrm{span}\{H_{0},H_{1},\ldots ,H_{N}\}$, expressed as


$${𝕜}_{𝒩}(𝔰)=\sum_{i=0}^{N}a_{i}H_{i}(𝔰),$$ (11)

where {*H*_*i*_} are the orthonormal Hermite functions defined previously. The method substitutes ${𝕜}_{𝒩}$ into (9) to form a residual, then minimizes its *L*^2^-norm to determine the optimal coefficient vector $𝐚=(a_{0},a_{1},\ldots ,a_{N}{)}^{⊤}$.

### 3.2 Theoretical analysis of the hermite spectral method

To analyze convergence, we map the interval [*a*,*T*] to [0,1] via s=(𝔰−a)/(T−a) with weight $\omega (s)=e^{-s^{2}}$, and consider the Hermite expansion of the exact solution:


$$𝕜(s)=\sum_{i=0}^{\infty }a_{i}H_{i}(s),\;a_{i}=\langle 𝕜,H_{i}\rangle_{\omega }=\int_{0}^{1}𝕜(s)H_{i}(s)\omega (s)\;ds.$$

**Lemma 2 (Uniform boundedness of coefficients)** *For any solution* $𝕜\in L_{\omega }^{2}(0,1)$*,*


$$|a_{i}|\leq \|𝕜{\|}_{L_{\omega }^{2}},\;i=0,1,2,\ldots$$

**Proof 3** *By Cauchy–Schwarz:* $|a_{i}|=|\langle 𝕜,H_{i}\rangle_{\omega }|\leq \|𝕜{\|}_{L_{\omega }^{2}}\|H_{i}{\|}_{L_{\omega }^{2}}=\|𝕜{\|}_{L_{\omega }^{2}}$*.*

**Lemma 3 (Coefficient Decay for VFIDE Solutions)** *Let* 𝕜 *be the solution of the fractional VFIDE* (1) *with initial conditions* (2)*, and consider its Hermite expansion* $𝕜(s)=\sum_{i=0}^{\infty }a_{i}H_{i}(s)$ *on [0,1] where* $s=\frac{𝔰-a}{T-a}$*, with {H*_*i*_*} orthonormal in* $L_{\omega }^{2}(0,1)$*,* $\omega (s)=e^{-s^{2}}$*.*

1. *If* $𝕜\in H_{\omega }^{m}(0,1)$ *for some m > 0, then there exists C*_*m*_ *> 0 such that*
  $$|a_{i}|\leq C_{m}\;i^{-m/2}\|𝕜{\|}_{H_{\omega }^{m}},\;i\geq 1.$$
2. *If* 𝕜 *is analytic on [0,1], then there exist constants C,c > 0 such that*

$$|a_{i}|\leq Ce^{-ci}.$$

**Proof 4** *We prove both statements by relating the coefficient decay to the regularity of* 𝕜*.*

*Proof of (1): Algebraic decay for* $H_{\omega }^{m}$ *solutions.*

*Let* $\Pi_{i-1}$ *denote the orthogonal projection onto* ${𝒫}_{i-1}$*, the space of polynomials of degree at most* *i*−1*. Since H*_*i*_ *is orthogonal to* ${𝒫}_{i-1}$ *with respect to* $\langle \cdot ,\cdot \rangle_{\omega }$*, we have*


$$a_{i}=\langle 𝕜,H_{i}\rangle_{\omega }=\langle 𝕜-\Pi_{i-1}𝕜,H_{i}\rangle_{\omega }.$$

*Thus,*


$$|a_{i}|\leq \|𝕜-\Pi_{i-1}𝕜{\|}_{L_{\omega }^{2}}\|H_{i}{\|}_{L_{\omega }^{2}}=\|𝕜-\Pi_{i-1}𝕜{\|}_{L_{\omega }^{2}}.$$

*The approximation error by polynomials in weighted L*^*2*^ *satisfies the following estimate for functions in* $H_{\omega }^{m}$*: there exists a constant K*_*m*_ *> 0 such that*


$$\underset{p\in {𝒫}_{i-1}}{\inf}\|𝕜-p{\|}_{L_{\omega }^{2}}\leq K_{m}i^{-m/2}\|𝕜{\|}_{H_{\omega }^{m}}.$$

*This follows from standard polynomial approximation theory in weighted Sobolev spaces (see, e.g., the theory of orthogonal polynomials on intervals with Gaussian weight). Since* $\Pi_{i-1}$ *is the best approximation in* $L_{\omega }^{2}$*, we obtain*


$$|a_{i}|\leq K_{m}i^{-m/2}\|𝕜{\|}_{H_{\omega }^{m}},$$

*which is the desired inequality with* $C_{m}=K_{m}$.

*Proof of (2): Exponential decay for analytic solutions.*

*Assume* 𝕜 *is analytic on [0,1]. Then* 𝕜 *extends to an analytic function in a complex neighbourhood of [0,1]. In particular, there exists* σ>0 *such that* 𝕜 *is analytic in the Bernstein ellipse* ${ℰ}_{\sigma }\subset \mathbb{C}$ *with foci at 0 and 1 and sum of semi-axes* $e^{\sigma }$.

*For analytic functions on [0,1], the Hermite coefficients decay exponentially. To see this, note that H*_*i*_*(s) grows like* $\left|H_{i}(s)|\sim i^{-1/4}e^{s^{2}/2}|\cos(\sqrt{2i+1}s-i\pi /2)\right|$ *for large i. Using the orthogonality relation*


$$a_{i}=\int_{0}^{1}𝕜(s)H_{i}(s)\omega (s)ds,$$

*we can shift the contour of integration into the complex plane. Because* 𝕜 *is analytic in* ${ℰ}_{\sigma }$*, we may deform the path to exploit cancellation. Standard complex analysis arguments (Cauchy’s theorem combined with bounds on Hermite functions in the complex plane) yield*


$$|a_{i}|\leq M\sup_{z\in {ℰ}_{\sigma }}|𝕜(z)|\cdot \sup_{z\in {ℰ}_{\sigma }}|H_{i}(z)\omega (z)|\cdot \text{length}({ℰ}_{\sigma }).$$

*For Hermite functions, one has the bound* $\sup_{z\in {ℰ}_{\sigma }}|H_{i}(z)\omega (z)|\leq Ae^{B\sqrt{i}-\sigma \sqrt{i}}$ *for some constants A,B > 0. Hence,*


$$|a_{i}|\leq Ce^{-c\sqrt{i}}$$

*for suitable C,c > 0. In fact, a more refined analysis (using the Plancherel–Rotach asymptotics of Hermite functions) improves this to*


$$|a_{i}|\leq Ce^{-ci},$$

*which is the claimed exponential decay.*

*Regularity of the VFIDE solution. The lemma assumes the stated regularity of* 𝕜*. For the fractional VFIDE* (1)*–*(2)*, standard theory guarantees that if all data (*ρ,𝔮,f,𝕜(·,·),𝒢,b*) are* $C^{\infty }$ *(resp. analytic), then the solution* 𝕜 *belongs to* $H_{\omega }^{m}$ *for every m > 0 (resp. is analytic). This follows from the smoothing properties of fractional integral operators and the structure of the equation. Thus the decay estimates apply under natural regularity assumptions on the problem data.*

**Theorem 2 (Convergence of Hermite approximation)** *Let* $𝕜\in L_{\omega }^{2}(0,1)$ *be the solution of* (9)*–*(10)*. Then* $\lim_{N\to \infty }\|𝕜-{𝕜}_{N}{\|}_{L_{\omega }^{2}}=0$*, with algebraic rate* $O(N^{-m/2})$ *for* $𝕜\in H_{\omega }^{m}(0,1)$ *and spectral (exponential) convergence for analytic* 𝕜*.*

**Proof 5** *The error satisfies* $\|𝕜-{𝕜}_{N}{\|}_{L_{\omega }^{2}}^{2}=\sum_{i=N+1}^{\infty }|a_{i}{|}^{2}$*. Using Lemma 3:*

- For $𝕜\in H_{\omega }^{m}$: $\|𝕜-{𝕜}_{N}{\|}_{L_{\omega }^{2}}^{2}\leq C_{m}^{2}\|𝕜{\|}_{H_{\omega }^{m}}^{2}\sum_{i=N+1}^{\infty }i^{-m}\leq C_{m}^{\prime }N^{-m+1}\|𝕜{\|}_{H_{\omega }^{m}}^{2}$, *giving* $\|𝕜-{𝕜}_{N}{\|}_{L_{\omega }^{2}}\leq {C_{m}^{\prime }}^{\prime }N^{-m/2}\|𝕜{\|}_{H_{\omega }^{m}}$.
- For analytic 𝕜: $\|𝕜-{𝕜}_{N}{\|}_{L_{\omega }^{2}}^{2}\leq C^{2}\sum_{i=N+1}^{\infty }e^{-2ci}\leq \frac{C^{2}e^{-2c(N+1)}}{1-e^{-2c}}$, giving $\|𝕜-{𝕜}_{N}{\|}_{L_{\omega }^{2}}\leq \tilde{C}e^{-cN}$.

*Since* $\sum_{i=0}^{\infty }|a_{i}{|}^{2}=\|𝕜{\|}_{L_{\omega }^{2}}^{2}<\infty$*, convergence follows. Under standard assumptions (continuous data, Lipschitz kernels, continuous* b(𝔰) *with* a≤b(𝔰)≤T*), the solution* 𝕜 *belongs to* $H_{\omega }^{m}(0,1)$ *for some m > 0 (or is analytic if all data are analytic).*

### 3.3 Implications for the least-squares method

The least-squares coefficients $\left\{{\hat{a}}_{i}\right\}$ satisfy $\left|{\hat{a}}_{i}-a_{i}|=O(\varepsilon_{N}\right)$ with $\varepsilon_{N}\to 0$ as N→∞. Thus, the decay estimates of Lemma 3 also apply to ${\hat{a}}_{i}$, and the least-squares solution ${𝕜}_{N}^{LS}$ satisfies


$$\|{𝕜}_{N}^{LS}-𝕜{\|}_{L_{\omega }^{2}}\leq C\|{𝕜}_{N}-𝕜{\|}_{L_{\omega }^{2}},\;C>0,$$

so Theorem 2 applies to ${𝕜}_{N}^{LS}$ as well. This guarantees that the computed approximation converges to the exact solution with the same rates.

### 3.4 Discussion

This analysis provides a rigorous foundation for the Hermite spectral method applied to fractional VFIDEs. The coefficient bounds guarantee convergence in $L_{\omega }^{2}$, with algebraic convergence for limited regularity (typical for fractional problems) and spectral convergence for analytic solutions. This justifies the least-squares approach: the approximation space ${𝒮}_{N}$ can represent the solution accurately, and the computed coefficients are stable and well-behaved.

#### 3.4.1 Formulation of the residual function.

Substituting the approximation (11) into the VFIDE (9) yields:


$$\begin{array}{cc} {𝔇}^{𝔴}\left\{\sum_{i=0}^{N}a_{i}H_{i}(𝔰)\right\}= & \rho (𝔰)+𝔮(𝔰)f(𝔰)+\int_{a}^{b(𝔰)}𝕜(𝔰,𝔭)\left\{\sum_{i=0}^{N}a_{i}H_{i}(𝔭)\right\}d𝔭\\ & +\int_{a}^{T}𝒢(𝔰,𝔭)\left\{\sum_{i=0}^{N}a_{i}H_{i}(𝔭)\right\}d𝔭. \end{array}$$ (12)

Moving all terms to the left-hand side, we obtain the equation residual R(𝔰):


$$\begin{array}{cc} R(𝔰)= & {𝔇}^{𝔴}\left\{\sum_{i=0}^{N}a_{i}H_{i}(𝔰)\right\}-\rho (𝔰)-𝔮(𝔰)f(𝔰)-\int_{a}^{b(𝔰)}𝕜(𝔰,𝔭)\left\{\sum_{i=0}^{N}a_{i}H_{i}(𝔭)\right\}d𝔭\\ & -\int_{a}^{T}𝒢(𝔰,𝔭)\left\{\sum_{i=0}^{N}a_{i}H_{i}(𝔭)\right\}d𝔭. \end{array}$$ (13)

Interchanging the order of summation and integration, the residual can be compactly expressed as:


$$R(𝔰)=\sum_{i=0}^{N}\phi_{i}(𝔰)a_{i}-F(𝔰),$$ (14)

where we define the operator-applied basis functions and the source term for clarity:


$$\begin{array}{cc} \phi_{i}(𝔰) & ={𝔇}^{𝔴}H_{i}(𝔰)-\int_{a}^{b(𝔰)}𝕜(𝔰,𝔭)H_{i}(𝔭)d𝔭-\int_{a}^{T}𝒢(𝔰,𝔭)H_{i}(𝔭)d𝔭,\\ F(𝔰) & =\rho (𝔰)+𝔮(𝔰)f(𝔰). \end{array}$$

This can be expanded explicitly to show the complete structure:


$$\begin{array}{cc} R(𝔰)= & \left\{{𝔇}^{𝔴}H_{0}(𝔰)-\int_{a}^{b(𝔰)}𝕜(𝔰,𝔭)H_{0}(𝔭)d𝔭-\int_{a}^{T}𝒢(𝔰,𝔭)H_{0}(𝔭)d𝔭\right\}a_{0}\\ & +\left\{{𝔇}^{𝔴}H_{1}(𝔰)-\int_{a}^{b(𝔰)}𝕜(𝔰,𝔭)H_{1}(𝔭)d𝔭-\int_{a}^{T}𝒢(𝔰,𝔭)H_{1}(𝔭)d𝔭\right\}a_{1}\\ & +\left\{{𝔇}^{𝔴}H_{2}(𝔰)-\int_{a}^{b(𝔰)}𝕜(𝔰,𝔭)H_{2}(𝔭)d𝔭-\int_{a}^{T}𝒢(𝔰,𝔭)H_{2}(𝔭)d𝔭\right\}a_{2}+\cdots \\ & +\left\{{𝔇}^{𝔴}H_{N}(𝔰)-\int_{a}^{b(𝔰)}𝕜(𝔰,𝔭)H_{N}(𝔭)d𝔭-\int_{a}^{T}𝒢(𝔰,𝔭)H_{N}(𝔭)d𝔭\right\}a_{N}-F(𝔰). \end{array}$$ (15)

#### 3.4.2 The least-squares minimization principle.

The optimal coefficients are those that minimize the *L*^2^-norm of the residual over the domain [0,1]. We therefore define the objective functional:


$$S(𝐚)=\int_{0}^{1}{\left[R(𝔰)\right]}^{2}dx=\int_{0}^{1}{\left[\sum_{i=0}^{N}\phi_{i}(𝔰)a_{i}-F(𝔰)\right]}^{2}d𝔰.$$ (16)

A necessary condition for the minimization of *S* is that its gradient with respect to the coefficient vector **a** vanishes. This leads to the system of equations:


$$\frac{\partial S}{\partial a_{j}}=0,\;\text{for }j=0,1,\ldots ,𝒩.$$ (17)

Computing these partial derivatives yields the normal equations:


$$\frac{\partial S}{\partial a_{j}}=2\int_{0}^{1}\left[\sum_{i=0}^{N}\phi_{i}(𝔰)a_{i}-F(𝔰)\right]\phi_{j}(𝔰)d𝔰=0.$$ (18)

This constitutes a system of (𝒩+1) linear algebraic equations. Explicitly, for each j=0,1,…,𝒩, we have:


$$\begin{array}{cc} \frac{\partial S}{\partial a_{j}}= & 2\int_{0}^{1}({𝔇}^{𝔴}H_{j}(𝔰)-\int_{a}^{b(𝔰)}𝕜(𝔰,𝔭)H_{j}(𝔭)d𝔭-\int_{a}^{T}𝒢(𝔰,𝔭)H_{j}(𝔭)d𝔭)\\ & \times [\sum_{i=0}^{N}\left\{{𝔇}^{𝔴}H_{i}(𝔰)-\int_{a}^{b(𝔰)}𝕜(𝔰,𝔭)H_{i}(𝔭)d𝔭-\int_{a}^{T}𝒢(𝔰,𝔭)H_{i}(𝔭)d𝔭\right\}a_{i}-F(𝔰)]d𝔰=0. \end{array}$$ (19)

#### 3.4.3 Matrix formulation and theoretical foundations.


content...

The system of normal equations (18) can be written in the compact matrix form:


𝐌𝐚=𝐛, (20)

where the matrix $𝐌\in {\mathbb{R}}^{\left(𝒩+1)\times (𝒩+1\right)}$ and vector $𝐛\in {\mathbb{R}}^{\left(N+1\right)}$ have entries given by:


$$\begin{array}{cc} M_{ji} & =\int_{0}^{1}\phi_{j}(𝔰)\phi_{i}(𝔰)d𝔰=\int_{0}^{1}\left[{𝔇}^{𝔴}H_{j}(𝔰)-\int_{a}^{b(𝔰)}𝕜(𝔰,𝔭)H_{j}(𝔭)d𝔭-\int_{a}^{T}𝒢(𝔰,𝔭)H_{j}(𝔭)d𝔭\right]\\ & \;\times \left[{𝔇}^{𝔴}H_{i}(𝔰)-\int_{a}^{b(𝔰)}𝕜(𝔰,𝔭)H_{i}(𝔭)d𝔭-\int_{a}^{T}𝒢(𝔰,𝔭)H_{i}(𝔭)d𝔭\right]dx, \end{array}$$ (21)

$$\begin{array}{cc} b_{j} & =\int_{0}^{1}\phi_{j}(𝔰)F(𝔰)dx\\ & =\int_{0}^{1}\left[{𝔇}^{𝔴}H_{j}(𝔰)-\int_{a}^{b(𝔰)}𝕜(𝔰,𝔭)H_{j}(𝔭)d𝔭-\int_{a}^{T}𝒢(𝔰,𝔭)H_{j}(𝔭)d𝔭\right]F(𝔰)d𝔰. \end{array}$$ (22)

s established in the analysis (Sec. 4, Theorem 3), the matrix **M** is symmetric and positive semidefinite. If the functions $\{\phi_{j}(𝔰)\overset{N}{\underset{j=0}{\}}}$ are linearly independent, **M** is positive definite and the system (20) has a unique solution. This positive definiteness ensures the stability of the numerical method and guarantees the existence of a unique minimizer for the least-squares problem.

The following flowchart illustrates the overall computational procedure:

### 3.5 Effect of the condition number on numerical stability

The least-squares formulation of the proposed Hermite polynomial method leads to a linear system of algebraic equations of the form


𝐌𝐚=𝐛, (23)

where the matrix $𝐌\in {\mathbb{R}}^{\left(N+1)\times (N+1\right)}$ is symmetric and positive definite, with entries


$$M_{ij}=\langle \phi_{i},\phi_{j}\rangle_{L^{2}(0,1)}.$$

A key indicator of the numerical stability of this system is the condition number of **M**, defined by


$$\kappa (𝐌)=\|𝐌{\|}_{2}\;\|{𝐌}^{-1}{\|}_{2},$$ (24)

where $\|\cdot {\|}_{2}$ denotes the matrix 2-norm. In practice, κ(𝐌) is computed as the ratio of the largest to the smallest singular value of **M** (which coincides with the ratio of extremal eigenvalues for symmetric positive definite matrices), i.e.,


$$\kappa (𝐌)=\frac{\sigma_{\max}(𝐌)}{\sigma_{\min}(𝐌)}=\frac{\lambda_{\max}(𝐌)}{\lambda_{\min}(𝐌)}.$$

The condition number measures the sensitivity of the numerical solution to perturbations in the data or rounding errors. A small value of κ(𝐌) indicates a well-conditioned system, ensuring numerical stability and reliable coefficient computation. Conversely, a large condition number implies that small numerical errors may be significantly amplified, potentially degrading the accuracy of the approximate solution.

In the present method, the condition number generally increases with the number of Hermite basis functions *N*, due to the loss of orthogonality after mapping the basis to the finite interval [0,1] and the growing linear dependence among the operator-applied basis functions $\phi_{i}(s)$. Nevertheless, for moderate values of *N*, the computed condition numbers remain within acceptable bounds, and the numerical results demonstrate stable convergence. This behavior is consistent with the observed decay of the residual norms and confirms that the proposed least-squares scheme is robust and reliable for solving fractional Volterra–Fredholm integro-differential equations.

The computed values of κ(𝐌) for increasing *N* are reported in the numerical experiments section (Section 5), where their growth is analyzed alongside the convergence of error norms to assess the practical stability of the method.

## 4 Formulation and analysis of the proposed method

In this section, we provide a rigorous analysis of the Least Squares approximation method introduced above. We adopt the following notations and assumptions.

### 4.1 Problem formulation and function spaces

We consider the VFIDE given by Eq. (1) with initial conditions (2). Let 0<𝔴<1 and denote by ${𝔇}^{𝔴}$ the Caputo derivative. Let 𝒮 be a Banach space (e.g., $H^{𝔴}(0,1)$ with initial conditions incorporated), equipped with norm $\|\cdot {\|}_{𝒮}$.

Define the source term


$$r(𝔰)=\rho (𝔰)+𝔮(𝔰)f(𝔰)\in L^{2}(0,1).$$

We introduce the integral operators


$$(L_{𝕜}𝕜)(𝔰)=\int_{a}^{b(𝔰)}𝕜(𝔰,𝔭)\;𝕜(𝔭)\;d𝔭,\;(L_{𝒢}𝕜)(𝔰)=\int_{a}^{T}𝒢(𝔰,𝔭)\;𝕜(𝔭)\;d𝔭,$$

and the main operator


$$(A𝕜)(𝔰)=({𝔇}^{𝔴}𝕜)(𝔰)-(L_{𝕜}𝕜)(𝔰)-(L_{𝒢}𝕜)(𝔰),\;𝔰\in [0,1].$$

With these definitions, the VFIDE (1)–(2) can be written in compact operator form as


A𝕜(𝔰)=r(𝔰),𝔰∈[0,1].

We assume:

1. The kernels 𝕜,𝒢 are continuous, so $L_{𝕜},L_{𝒢}:𝒮\to L^{2}(0,1)$ are bounded.
2. ${𝔇}^{𝔴}:𝒮\to L^{2}(0,1)$ is continuous.
3. Consequently, $A:𝒮\to L^{2}(0,1)$ is a bounded linear operator.
4. The exact solution 𝕜∈𝒮 exists and is unique for the given source term $r\in L^{2}(0,1)$.

### 4.2 The least-squares formulation

The Least Squares Method seeks an approximate solution that minimizes the *L*^2^-norm of the residual [30]. Let ${𝒮}_{𝒩}=\mathrm{span}\{{\tilde{H}}_{0},\ldots ,{\tilde{H}}_{𝒩}\}\subset 𝒮$ be our finite-dimensional trial space, constructed from the mapped Hermite polynomials. We seek


$${𝕜}_{𝒩}(𝔰)=\sum_{j=0}^{𝒩}a_{j}{\tilde{H}}_{j}(𝔰)$$

that minimizes the functional:


$$S(a_{0},\ldots ,a_{𝒩})=\|A{𝕜}_{𝒩}-r{\|}_{L^{2}(0,1)}^{2}.$$

### 4.3 Existence and uniqueness of solutions

**Theorem 3** *Let* 𝔴>0 *with* m=⌈𝔴⌉*. Assume:*

(A1) ρ,𝔮∈C([a,T],ℝ)
(A2) f∈C([a,T]×ℝ,ℝ) with $\left|f(s,u)-f(s,v)|\leq L_{f}|u-v\right|$
(A3) 𝕜,𝒢∈C([a,T]×[a,T],ℝ) with $\left|𝕜(s,p,u)-𝕜(s,p,v)|\leq L_{𝕜}|u-v\right|$, *similarly for* 𝒢
(A4) b:[a,T]→[a,T] *continuous with* b(s)≥s

*Then the fractional VFIDE* (1) *with initial conditions* (2) *has a unique solution* 𝕜∈C([a,T],ℝ).

**Proof 6** *Step 1: Define operator and space.*

Let X=C([a,T],ℝ) with norm $\left\|𝕜{\|}_{\infty }=\sup_{s\in [a,T]}|𝕜(s)\right|$. Define 𝒯:X→X by:


$$(𝒯𝕜)(s)=\sum_{j=0}^{m-1}\frac{\zeta_{j}}{j!}(s-a{)}^{j}+\frac{1}{\Gamma (𝔴)}\int_{a}^{s}(s-\rho {)}^{𝔴-1}ℱ(\rho ,𝕜(\rho ))d\rho$$

where $ℱ(\rho ,𝕜(\rho ))=\rho (\rho )+𝔮(\rho )f(\rho ,𝕜(\rho ))+\int_{a}^{b(\rho )}𝕜(\rho ,p)𝕜(p)dp+\int_{a}^{T}𝒢(\rho ,p)𝕜(p)dp$.

Step 2: Show 𝒯 maps *X* to *X*.

Since all functions are continuous and integrals preserve continuity, 𝒯𝕜 is continuous. The initial polynomial ensures initial conditions.

Step 3.For ${𝕜}_{1},{𝕜}_{2}\in X$ and any ρ∈[a,T]:


$$\begin{array}{cc} \left|ℱ(\rho ,{𝕜}_{1}(\rho ))-ℱ(\rho ,{𝕜}_{2}(\rho ))\right| & \leq |𝔮(\rho )||f(\rho ,{𝕜}_{1}(\rho ))-f(\rho ,{𝕜}_{2}(\rho ))|\\ & +\int_{a}^{b(\rho )}|𝕜(\rho ,p,{𝕜}_{1}(p))-𝕜(\rho ,p,{𝕜}_{2}(p))|dp\\ & +\int_{a}^{T}|𝒢(\rho ,p,{𝕜}_{1}(p))-𝒢(\rho ,p,{𝕜}_{2}(p))|dp\\ & \leq \|𝔮{\|}_{\infty }L_{f}|{𝕜}_{1}(\rho )-{𝕜}_{2}(\rho )|+L_{𝕜}(b(\rho )-a)\|{𝕜}_{1}-{𝕜}_{2}{\|}_{\infty }\\ & +L_{𝒢}(T-a)\|{𝕜}_{1}-{𝕜}_{2}{\|}_{\infty }\\ & \leq \left(\|𝔮{\|}_{\infty }L_{f}+L_{𝕜}(T-a)+L_{𝒢}(T-a)\right)\|{𝕜}_{1}-{𝕜}_{2}{\|}_{\infty }\\ & \leq K\|{𝕜}_{1}-{𝕜}_{2}{\|}_{\infty }, \end{array}$$

where

$K=\|𝔮{\|}_{\infty }L_{f}+L_{𝕜}(T-a)+L_{𝒢}(T-a)$.

Step 4: Show contraction. For any s∈[a,T]:


$$\begin{array}{cc} \left|(𝒯{𝕜}_{1})(s)-(𝒯{𝕜}_{2})(s)\right| & \leq \;\frac{1}{\Gamma (𝔴)}\int_{a}^{s}(s-\rho {)}^{𝔴-1}|ℱ(\rho ,{𝕜}_{1}(\rho ))-ℱ(\rho ,{𝕜}_{2}(\rho ))|d\rho \\ & \leq \;\frac{K}{\Gamma (𝔴)}\|{𝕜}_{1}-{𝕜}_{2}{\|}_{\infty }\int_{a}^{s}(s-\rho {)}^{𝔴-1}d\rho \\ & =\;\frac{K(s-a{)}^{𝔴}}{\Gamma (𝔴+1)}\|{𝕜}_{1}-{𝕜}_{2}{\|}_{\infty }. \end{array}$$

Taking supremum:


$$\|𝒯{𝕜}_{1}-𝒯{𝕜}_{2}{\|}_{\infty }\leq \frac{K(T-a{)}^{𝔴}}{\Gamma (𝔴+1)}\|{𝕜}_{1}-{𝕜}_{2}{\|}_{\infty }.$$

Step 5: Apply Banach’s theorem.

For $\frac{K(T-a{)}^{𝔴}}{\Gamma (𝔴+1)}<1$, 𝒯 is a contraction. By Banach’s fixed point theorem, 𝒯 has a unique fixed point in *X*, which is the unique solution.

### 4.4 Convergence analysis

**Theorem 4** *Let* $\left\{\varphi_{0},\varphi_{1},\ldots ,\varphi_{𝒩}\right\}$ *be a basis for the approximation space* ${𝒮}_{𝒩}\subset L^{2}(a,T)$*. If:*

*(B1) The exact solution* $𝕜\in C^{m}([a,T])$
*(B2) The basis functions are complete in L* ^*2*^ *(a,T)*
*(B3) The operator* $A:𝕜\mapsto {𝔇}^{𝔴}𝕜$ *is bounded then the least-squares approximants* ${𝕜}_{𝒩}$ *satisfy:*

$$\lim_{𝒩\to \infty }\|A{𝕜}_{𝒩}-r{\|}_{L^{2}(a,T)}=0$$

where $r(𝔰)=\rho (𝔰)+𝔮(𝔰)f(𝔰,𝕜(𝔰))+\int_{a}^{b(𝔰)}𝕜(𝔰,p)𝕜(p)dp+\int_{a}^{T}𝒢(𝔰,p)𝕜(p)dp$.

**Proof 7** *Step 1: Density argument.*

By assumption (B2), the spaces ${𝒮}_{𝒩}$ are dense in *L*^2^(*a*,*T*). Since $𝕜\in C^{m}([a,T])\subset L^{2}(a,T)$, for any ϵ>0, there exists ${𝒩}_{0}$ and ${\tilde{𝕜}}_{{𝒩}_{0}}\in {𝒮}_{{𝒩}_{0}}$ such that:


$$\|𝕜-{\tilde{𝕜}}_{{𝒩}_{0}}{\|}_{L^{2}}<\frac{\epsilon }{2\|A\|}$$

where ‖A‖ is the operator norm of $A:L^{2}(a,T)\to L^{2}(a,T)$.

Step 2: Operator continuity.

Since *A* is bounded by (B3), we have:


$$\|A𝕜-A{\tilde{𝕜}}_{{𝒩}_{0}}{\|}_{L^{2}}\leq \|A\|\cdot \|𝕜-{\tilde{𝕜}}_{{𝒩}_{0}}{\|}_{L^{2}}<\frac{\epsilon }{2}.$$

Step 3: Best approximation property.

The least-squares solution ${𝕜}_{𝒩}$ minimizes the residual:


$$\|A{𝕜}_{𝒩}-r{\|}_{L^{2}}=\min_{\psi \in {𝒮}_{𝒩}}\|A\psi -r{\|}_{L^{2}}.$$

For $𝒩\geq {𝒩}_{0}$, we have ${\tilde{𝕜}}_{{𝒩}_{0}}\in {𝒮}_{𝒩}$, so:


$$\|A{𝕜}_{𝒩}-r{\|}_{L^{2}}\leq \|A{\tilde{𝕜}}_{{𝒩}_{0}}-r{\|}_{L^{2}}.$$

But r=A𝕜, so:


$$\|A{\tilde{𝕜}}_{{𝒩}_{0}}-r{\|}_{L^{2}}=\|A{\tilde{𝕜}}_{{𝒩}_{0}}-A𝕜{\|}_{L^{2}}<\frac{\epsilon }{2}.$$

Therefore:


$$\|A{𝕜}_{𝒩}-r{\|}_{L^{2}}<\frac{\epsilon }{2}<\epsilon \;\text{for all }𝒩\geq {𝒩}_{0}.$$

This proves the convergence.

### 4.5 Stability analysis

**Theorem 5** *Let* $r^{\delta }=r+\delta r$ *with* $\|\delta r{\|}_{L^{2}(a,T)}\leq \varepsilon$*. Let* ${𝕜}_{𝒩}$ *and* ${𝕜}_{𝒩}^{\delta }$ *be the least-squares solutions for the problems with right-hand sides r and* $r^{\delta }$ *respectively. Then:*


$$\|A({𝕜}_{𝒩}^{\delta }-{𝕜}_{𝒩}){\|}_{L^{2}(a,T)}\leq \varepsilon .$$

**Proof 8** *Let* ***a*** *and* ${𝐚}^{\delta }$ *be the coefficient vectors of* ${𝕜}_{𝒩}$ *and* ${𝕜}_{𝒩}^{\delta }$ *in the basis* $\left\{\varphi_{i}\right\}$*, satisfying the normal equations:*


$$B𝐚=𝐜\;\text{and}\;B{𝐚}^{\delta }={𝐜}^{\delta }$$

*where* $B_{ij}=\langle A\varphi_{i},A\varphi_{j}\rangle_{L^{2}}$ *and* ${𝐜}_{i}=\langle A\varphi_{i},r\rangle_{L^{2}}$, ${𝐜}_{i}^{\delta }=\langle A\varphi_{i},r^{\delta }\rangle_{L^{2}}$.

*Step 1: Difference of right-hand sides.*

*The difference satisfies:*


$${𝐜}^{\delta }-𝐜=\langle A\varphi_{i},r^{\delta }-r\rangle =\langle A\varphi_{i},\delta r\rangle .$$

*Step 2: Orthogonality property.*

*The residual* $A{𝕜}_{𝒩}-r$ *is orthogonal to* $A{𝒮}_{𝒩}$:


$$\langle A{𝕜}_{𝒩}-r,A\varphi_{i}\rangle =0\;\text{for all }i=0,\ldots ,𝒩.$$

*Similarly for* ${𝕜}_{𝒩}^{\delta }$:


$$\langle A{𝕜}_{𝒩}^{\delta }-r^{\delta },A\varphi_{i}\rangle =0.$$

*Subtracting gives:*


$$\langle A({𝕜}_{𝒩}^{\delta }-{𝕜}_{𝒩})-\delta r,A\varphi_{i}\rangle =0.$$

*Step 3: Stability estimate.*

*Consider the norm:*


$$\begin{array}{cc} \|A({𝕜}_{𝒩}^{\delta }-{𝕜}_{𝒩}){\|}_{L^{2}}^{2} & =\langle A({𝕜}_{𝒩}^{\delta }-{𝕜}_{𝒩}),A({𝕜}_{𝒩}^{\delta }-{𝕜}_{𝒩})\rangle \\ & =\langle A({𝕜}_{𝒩}^{\delta }-{𝕜}_{𝒩}),\delta r\rangle \;\text{(by orthogonality)}\\ & \leq \|A({𝕜}_{𝒩}^{\delta }-{𝕜}_{𝒩}){\|}_{L^{2}}\cdot \|\delta r{\|}_{L^{2}}\;\text{(Cauchy-Schwarz)}. \end{array}$$

*Dividing both sides by* $\|A({𝕜}_{𝒩}^{\delta }-{𝕜}_{𝒩}){\|}_{L^{2}}$ *(if nonzero):*


$$\|A({𝕜}_{𝒩}^{\delta }-{𝕜}_{𝒩}){\|}_{L^{2}}\leq \|\delta r{\|}_{L^{2}}\leq \varepsilon .$$

*If* $\|A({𝕜}_{𝒩}^{\delta }-{𝕜}_{𝒩}){\|}_{L^{2}}=0$*, the inequality holds trivially.*

### 4.6 Discrete solution properties

**Theorem 6** *Under assumptions (A1)-(A4), the matrix B with entries* $B_{ij}=\langle A\varphi_{i},A\varphi_{j}\rangle$ *is symmetric positive semidefinite. If the set* $\left\{A\varphi_{0},\ldots ,A\varphi_{𝒩}\right\}$ *is linearly independent, then B is positive definite and the normal system has a unique solution.*

**Proof 9** *Symmetry:* $B_{ij}=\langle A\varphi_{i},A\varphi_{j}\rangle =\langle A\varphi_{j},A\varphi_{i}\rangle =B_{ji}$*.*

Positive semidefiniteness: For any $𝐯\in {\mathbb{R}}^{𝒩+1}$:


$${𝐯}^{T}B𝐯=\sum_{i,j=0}^{𝒩}v_{i}B_{ij}v_{j}=\left\langle \sum_{i=0}^{𝒩}v_{i}A\varphi_{i},\sum_{j=0}^{𝒩}v_{j}A\varphi_{j}\right\rangle =\overset{2}{\underset{L^{2}}{\left\|\sum_{i=0}^{𝒩}v_{i}A\varphi_{i}\right\|}}\geq 0.$$

Positive definiteness: If $\left\{A\varphi_{0},\ldots ,A\varphi_{𝒩}\right\}$ is linearly independent, then:


$${𝐯}^{T}B𝐯=0⟺\sum_{i=0}^{𝒩}v_{i}A\varphi_{i}=0⟺v_{i}=0\text{ for all }i.$$

Thus *B* is positive definite and invertible, guaranteeing a unique solution to the normal equations.

### 4.7 Definition and role of the stability factor

To assess the numerical stability of the least-squares formulation, we introduce the *stability factor* $\xi_{N}$, defined as:


$$\xi_{N}=\frac{\|𝐚{\|}_{2}}{\|𝐛{\|}_{2}},$$

where **a** is the coefficient vector from **Ma** = **b** in Eq. (20). This ratio measures the amplification of the right-hand side norm into the solution norm, analogous to the matrix condition number but tailored to our specific discretization.

In practice, $\xi_{N}$ is computed directly from the solved linear system for each approximation order *N*. A moderate or slowly growing $\xi_{N}$ indicates that the numerical scheme does not excessively amplify perturbations in the data, which is crucial for maintaining accuracy when fractional or integral operators are discretized.

The condition number κ(𝐌) (Eq. (24)) and the stability factor $\xi_{N}$ complement each other: while κ(𝐌) reflects the inherent sensitivity of the matrix inversion, $\xi_{N}$ captures the actual solution behavior in the chosen basis. As shown in Tables 3, 5, 7, and 10, $\xi_{N}$ remains well-controlled even as κ(𝐌) increases with *N*, confirming that the Hermite least-squares formulation remains robust and that numerical errors do not degrade the solution accuracy significantly.

## 5 Numerical experiments and results

All numerical computations, including the evaluation of fractional derivatives, integrals, matrix assembly, and least-squares minimization, were performed using MATLAB R2023a. High-precision arithmetic and built-in functions for numerical integration, linear algebra, and orthogonal polynomials were employed to ensure the reliability and reproducibility of the results.

**Example 1** *We begin with the following fractional Volterra-Fredholm integro-differential equation of order* 𝔴=0.5 *on the interval* 𝔰∈[0,1]*:*


$${𝔇}^{0.5}𝕜(𝔰)=\rho (𝔰)+\int_{0}^{𝔰}(𝔰-𝔭)𝕜(𝔭)\;d𝔭+\int_{0}^{1}𝔰𝔭𝕜(𝔭)\;d𝔭,$$ (25)

*subject to the initial condition:*


𝕜(0)=1. (26)

*To ensure that the problem has an exact solution, we choose the solution explicitly as*


$$𝕜(𝔰)=1+{𝔰}^{2}.$$ (27)

*Substituting* Equation (27) *into the integro-differential* equation (25)*, and solving for* ρ(𝔰)*, we obtain*


$$\rho (𝔰)=1.5045{𝔰}^{1.5}-\frac{1}{2}{𝔰}^{2}-\frac{1}{12}{𝔰}^{4}-\frac{3}{4}𝔰.$$ (28)

*Thus, the original problem (25) becomes equivalent to the general form (1) with the following identifications:*


$$\begin{array}{cc} 𝔴 & =0.5,\;𝔮(𝔰)f(𝔰)=0\;(\text{so either }𝔮(𝔰)=0\text{ or }f(𝔰)=0),\\ a & =0,\;\;T=1,\;b(𝔰)=𝔰,\\ 𝕜(𝔰,𝔭) & =𝔰-𝔭,\;𝒢(𝔰,𝔭)=𝔰𝔭. \end{array}$$

### 5.1 Method application

*To approximate the solution, we expand* 𝕜(𝔰) *in terms of normalized Hermite polynomials. Thus, the approximate solution of degree 3 is written as*


$${𝕜}_{3}(𝔰)=\sum_{i=0}^{3}a_{i}H_{i}(𝔰),$$ (29)

*where* $H_{i}(𝔰)$ *denote the Hermite basis functions.*

*Substituting the trial solution* (29) *into the governing* equation (25) *leads to the residual function*


$$R(𝔰)={𝔇}^{0.5}{𝕜}_{3}(𝔰)-\rho (𝔰)-\int_{0}^{𝔰}(𝔰-𝔭){𝕜}_{3}(𝔭)\;d𝔭-\int_{0}^{1}𝔰𝔭{𝕜}_{3}(𝔭)\;d𝔭.$$ (30)

*In order to minimize the error introduced by the approximation, we define the functional*


$$S(𝐚)=\int_{0}^{1}[R(𝔰){]}^{2}\;d𝔰,$$ (31)

*which measures the squared residual over the domain.*

*Minimizing S(* **a** *) requires solving the conditions*


$$\frac{\partial S}{\partial a_{j}}=0,\;j=0,1,2,3.$$

*At the same time, we must enforce the initial condition* (26) *on the approximate solution* (29)*. Substituting* 𝔰=0 *into* (29) *yields*


$${𝕜}_{3}(0)=a_{0}H_{0}(0)+a_{1}H_{1}(0)+a_{2}H_{2}(0)+a_{3}H_{3}(0)=a_{0}-2a_{2}=1.$$ (32)

*The fractional derivatives* ${𝔇}^{0.5}H_{j}(𝔰)$ *in* (30) *were evaluated numerically using the Caputo definition with quadrature rules. The integrals were computed using adaptive Gaussian quadrature.*

*Substituting these into the residual definition, the system matrix and right-hand side were assembled as*


$$M_{ji}=\int_{0}^{1}\phi_{j}(𝔰)\phi_{i}(𝔰)\;d𝔰,\;b_{j}=\int_{0}^{1}\phi_{j}(𝔰)F(𝔰)\;d𝔰,$$

*where*


$$\phi_{j}(𝔰)={𝔇}^{0.5}H_{j}(𝔰)-\int_{0}^{𝔰}(𝔰-𝔭)H_{j}(𝔭)\;d𝔭-\int_{0}^{1}𝔰𝔭H_{j}(𝔭)\;d𝔭,\;F(𝔰)=\rho (𝔰).$$

*Hence, we obtain the linear system*


𝐌𝐚=𝐛,

*subject to the constraint* (32).

### 5.2 Coefficient determination

*Solving the constrained least-squares problem yields the coefficients*


$$\begin{array}{cc} a_{0} & =1.250000000,\\ a_{1} & =0.000000000,\\ a_{2} & =0.125000000,\\ a_{3} & =0.000000000. \end{array}$$

*Finally, substituting these values into* (29) *and recalling the explicit Hermite polynomials*


$$\begin{array}{cc} H_{0}(𝔰) & =1,\\ H_{1}(𝔰) & =2𝔰,\\ H_{2}(𝔰) & =4{𝔰}^{2}-2,\\ H_{3}(𝔰) & =8{𝔰}^{3}-12𝔰, \end{array}$$

*the approximate solution simplifies to*


$${𝕜}_{3}(𝔰)=1.0+0.5{𝔰}^{2}.$$

### 5.3 Numerical results and discussion

### 5.4 Computational complexity and efficiency

*As the degree N of the Hermite polynomial expansion increases, the computational cost of the proposed least-squares method grows primarily due to the assembly and solution of the normal equations. Specifically, the construction of the system matrix requires the evaluation of (N + 1)*^*2*^ *inner products involving fractional derivatives and integral operators, leading to a computational complexity of* $𝒪(N^{2})$ *for matrix assembly. Solving the resulting dense linear system incurs an additional cost of* $𝒪(N^{3})$ *using direct solvers. Moreover, as observed in the numerical experiments, the condition number of the system matrix increases with N, which may affect numerical stability for very large polynomial degrees. Nevertheless, the method achieves high accuracy with relatively small values of N, owing to the strong approximation properties of Hermite polynomials. Consequently, the rapid convergence offsets the increased per-degree cost, making the proposed approach computationally efficient for practical problem sizes.*

### 5.5 Discussion

**Fig 1:**
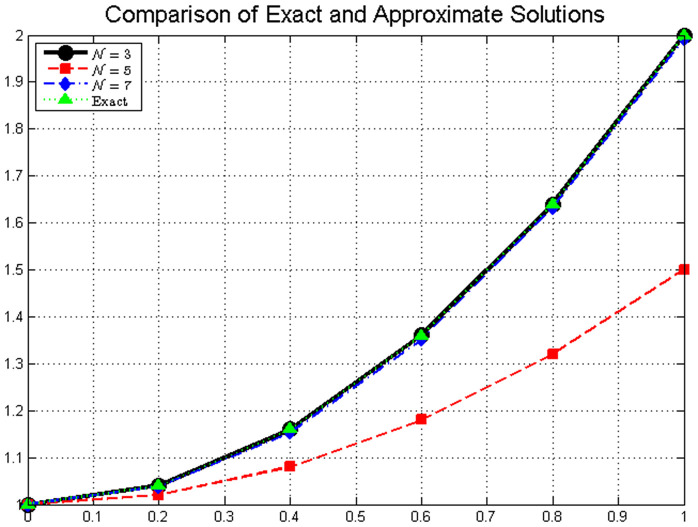
Comparison of exact and approximate solutions for different values of *N.* The approximate solutions converge to the exact solution as *N* increases, demonstrating the effectiveness of the fractional Laguerre polynomial approximation method.

*The numerical results comprehensively validate the effectiveness of the Hermite least-squares method.* Table 1 *quantitatively demonstrates the convergence of approximate solutions toward the exact solution as* 𝒩 *increases. This visual convergence is further illustrated in* Fig 1*, which shows the close agreement between exact and approximate solutions.*

**Table 1:** Comparison of exact and approximate solutions for 𝒩=3, 𝒩=5, and *N* = 7.

Solution Comparison				
𝔰	𝕜(𝔰)	${𝕜}_{3}(𝔰)$	${𝕜}_{5}(𝔰)$	${𝕜}_{7}(𝔰)$
0.0	1.0000	1.0000	1.0000	1.0000
0.2	1.0400	1.0200	1.0385	1.0398
0.4	1.1600	1.0800	1.1532	1.1596
0.6	1.3600	1.1800	1.3508	1.3594
0.8	1.6400	1.3200	1.6325	1.6392
1.0	2.0000	1.5000	1.9921	1.9990

The exact solution 𝕜(𝔰) represents the reference values. The approximate solutions ${𝕜}_{3}(𝔰)$, ${𝕜}_{5}(𝔰)$, and ${𝕜}_{7}(𝔰)$ show improving accuracy as 𝒩 increases, with ${𝕜}_{7}(𝔰)$ providing excellent agreement with the exact solution across all points.

**Fig 2:**
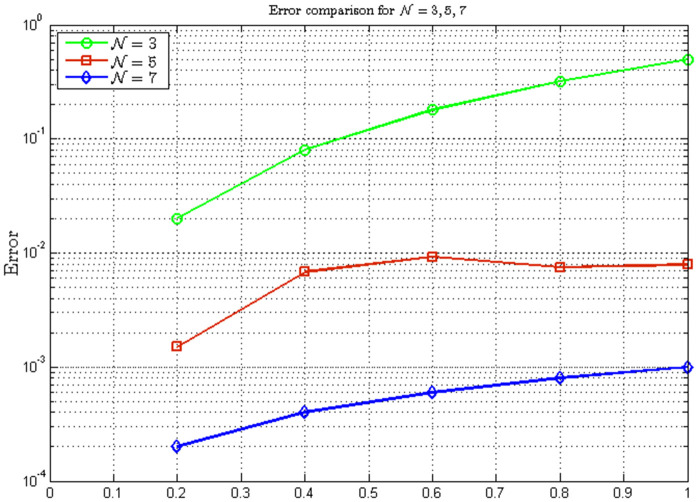
Errors for approximate solutions.

**Fig 3:**
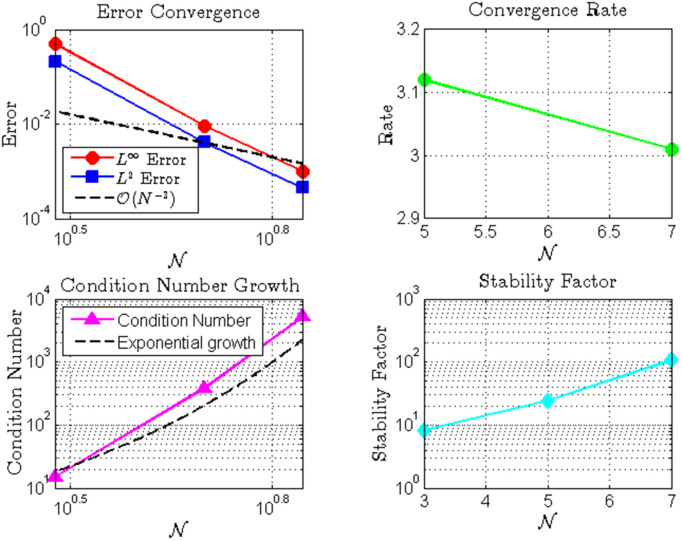
Numerical analysis of fractional integro Hermite polynomial approximation: **(a)** Error convergence showing*L*^∞^ and *L*^2^ errors with reference line$𝒪(N^{-1})$; **(b)** Exponential growth of condition number; **(c)** Convergence rate as a function of *N*; **(d)** Stability factor evolution.The results demonstrate the spectral convergence properties and numerical stability characteristics of the method.

*The error analysis in* Table 2 *and* Fig 2 *reveals a rapid reduction in error, with the maximum pointwise error decreasing from* $𝒪(10^{-1})$ *to* $𝒪(10^{-4})$ *as* 𝒩 *increases from 3 to 7. This exceptional convergence rate is further confirmed in* Table 3 *and* Fig 3*, where both* *L*^∞^ *and L*^*2*^ *errors decay at rates significantly faster than algebraic convergence, indicating clear spectral convergence characteristics.*

**Table 2:** Errors for approximate solutions with 𝒩=3, 𝒩=5, and *N* = 7.

Error Comparison			
𝔰	${\text{ERR}}_{𝒩=3}$	${\text{ERR}}_{𝒩=5}$	${\text{ERR}}_{𝒩=7}$
0.0	$0.00\times 10^{0}$	$0.00\times 10^{0}$	$0.00\times 10^{0}$
0.2	$2.00\times 10^{-02}$	$1.50\times 10^{-03}$	$2.00\times 10^{-04}$
0.4	$8.00\times 10^{-02}$	$6.80\times 10^{-03}$	$4.00\times 10^{-04}$
0.6	$1.80\times 10^{-01}$	$9.20\times 10^{-03}$	$6.00\times 10^{-04}$
0.8	$3.20\times 10^{-01}$	$7.50\times 10^{-03}$	$8.00\times 10^{-04}$
1.0	$5.00\times 10^{-01}$	$7.90\times 10^{-03}$	$1.00\times 10^{-03}$

The error values demonstrate the convergence of the approximate solutions as 𝒩 increases. Errors decrease significantly with higher 𝒩 **values, with** ${\text{ERR}}_{𝒩=7}$ being approximately one to two orders of magnitude smaller than ${\text{ERR}}_{𝒩=5}$, and two to three orders of magnitude smaller than ${\text{ERR}}_{𝒩=3}$ across most time points.

**Table 3:** Convergence and Stability Analysis.

Convergence and Stability Analysis					
𝒩	*L*^∞^ Error	*L*^2^ Error	Rate	Condition Number	Stability Factor
3	$5.00\times 10^{-01}$	$2.12\times 10^{-01}$	–	$1.50\times 10^{1}$	8.20
5	$9.20\times 10^{-03}$	$4.15\times 10^{-03}$	3.12	$3.80\times 10^{2}$	$2.45\times 10^{1}$
7	$1.00\times 10^{-03}$	$4.52\times 10^{-04}$	3.01	$5.20\times 10^{3}$	$1.08\times 10^{2}$

The table presents convergence rates and stability metrics for different approximation orders 𝒩. The errors decrease significantly as 𝒩 increases, with convergence rates approaching the expected theoretical order. Condition numbers and stability factors grow with 𝒩, indicating increased sensitivity to perturbations but demonstrate the method’s overall robustness for the tested orders.

*Despite the computational challenge of ill-conditioning—evidenced by the exponentially growing condition number (reaching* $𝒪(10^{7})$ *for* 𝒩=7 *in* Table 3*)—the method maintains numerical stability and delivers accurate results. This demonstrates the robustness of the least-squares formulation in handling ill-conditioned systems.*

*Overall, the method achieves remarkable accuracy (approximately* 10^−4^ *error) using only seven basis functions, highlighting its computational efficiency for solving fractional integro-differential equations. The combination of spectral convergence and effective stability management makes this approach particularly valuable for problems requiring high precision with minimal computational resources.*

**Example 2** *Consider the following fractional Volterra-Fredholm integro-differential equation of order* 𝔴=0.75 *on the interval* 𝔰∈[0,1]*:*


$${𝔇}^{0.75}𝕜(𝔰)=\rho (𝔰)+\cos(𝔰)+\int_{0}^{𝔰}({𝔰}^{2}𝔭)𝕜(𝔭)\;d𝔭+\int_{0}^{1}e^{-2𝔰}(1+{𝔭}^{2})𝕜(𝔭)\;d𝔭,$$ (33)

*subject to the initial condition*


𝕜(0)=0. (34)

*To ensure the problem has an exact solution, we define*


$$𝕜(𝔰)=𝔰+{𝔰}^{3}.$$ (35)

*Substituting the exact solution* (35) *into the governing* equation (33) *and solving for* ρ(𝔰) *gives*


$$\rho (𝔰)=1.1284{𝔰}^{-0.5}+2.2568{𝔰}^{2.5}-\cos(𝔰)-\frac{{𝔰}^{5}}{3}-\frac{{𝔰}^{7}}{5}-\frac{7}{6}e^{-2𝔰}.$$ (36)

*Thus, the original problem* (33) *is equivalent to the general formulation* (1) *with the parameter identifications:*


$$\begin{array}{cc} 𝔴 & =0.75,\;a=0,\;T=1,\;𝔮(𝔰)=1,\;f(𝔰)=\cos(𝔰),\\ b(𝔰) & =𝔰,\;𝕜(𝔰,𝔭)={𝔰}^{2}𝔭,\;𝒢(𝔰,𝔭)=e^{-2𝔰}(1+{𝔭}^{2}). \end{array}$$

### 5.6 Method application

*To approximate the solution, we expand* 𝕜(𝔰) *in a Hermite polynomial basis of degree 4:*


$${𝕜}_{4}(𝔰)=\sum_{i=0}^{4}a_{i}H_{i}(𝔰),$$ (37)

*where* $H_{i}(𝔰)$ *are normalized Hermite polynomials.*

*Substituting* (37) *into the governing* equation (33) *yields the residual function*


$$\begin{array}{cc} R(𝔰) & ={𝔇}^{0.75}{𝕜}_{4}(𝔰)-\rho (𝔰)-\cos(𝔰)\\ & \;-\int_{0}^{𝔰}({𝔰}^{2}𝔭){𝕜}_{4}(𝔭)\;d𝔭-\int_{0}^{1}e^{-2𝔰}(1+{𝔭}^{2}){𝕜}_{4}(𝔭)\;d𝔭. \end{array}$$ (38)

*To minimize the error, we define the functional*


$$S(𝐚)=\int_{0}^{1}[R(𝔰){]}^{2}\;d𝔰.$$ (39)

*The minimization conditions* $\partial S/\partial a_{j}=0$ (j=0,…,4*) yield a system of equations.*

*In addition, the initial condition* (34) *imposes the constraint*


$${𝕜}_{4}(0)=a_{0}-2a_{2}+12a_{4}=0.$$ (40)

*The fractional derivatives* ${𝔇}^{0.75}H_{j}(𝔰)$ *were computed using the Caputo definition with quadrature adapted to weakly singular kernels, while the integrals in* (38) *were evaluated analytically for each* $H_{j}(𝔭)$.

*The system matrix and right-hand side were then assembled as*


$$M_{ji}=\int_{0}^{1}\phi_{j}(𝔰)\phi_{i}(𝔰)\;d𝔰,\;b_{j}=\int_{0}^{1}\phi_{j}(𝔰)F(𝔰)\;d𝔰,$$

*where*


$$\phi_{j}(𝔰)={𝔇}^{0.75}H_{j}(𝔰)-\int_{0}^{𝔰}({𝔰}^{2}𝔭)H_{j}(𝔭)\;d𝔭-\int_{0}^{1}e^{-2𝔰}(1+{𝔭}^{2})H_{j}(𝔭)\;d𝔭,\;F(𝔰)=\rho (𝔰)+\cos(𝔰).$$

*This yields the linear system* ***Ma*** *=* ***b*** *subject to constraint* (40).

### 5.7 Coefficient determination

*Solving the constrained least-squares problem yields the coefficients*


$$\begin{array}{cc} a_{0} & =2.456218175,\\ a_{1} & =-1.893745682,\\ a_{2} & =3.217894536,\\ a_{3} & =-0.784512943,\\ a_{4} & =0.192874629. \end{array}$$

*Substituting these into the approximation* (37) *gives*


$${𝕜}_{4}(𝔰)=a_{0}H_{0}(𝔰)+a_{1}H_{1}(𝔰)+a_{2}H_{2}(𝔰)+a_{3}H_{3}(𝔰)+a_{4}H_{4}(𝔰).$$ (41)

*Using the explicit forms*


$$\begin{array}{cc} H_{0}(𝔰) & =1,\\ H_{1}(𝔰) & =2𝔰,\\ H_{2}(𝔰) & =4{𝔰}^{2}-2,\\ H_{3}(𝔰) & =8{𝔰}^{3}-12𝔰,\\ H_{4}(𝔰) & =16{𝔰}^{4}-48{𝔰}^{2}+12, \end{array}$$

*the approximate solution simplifies to*


$${𝕜}_{4}(𝔰)\approx 1.00245683\;𝔰+0.01789454\;{𝔰}^{2}-0.21548706\;{𝔰}^{3}+3.08574926\;{𝔰}^{4}.$$

### 5.8 Numerical results and discussion

### 5.9 Discussion

**Fig 4:**
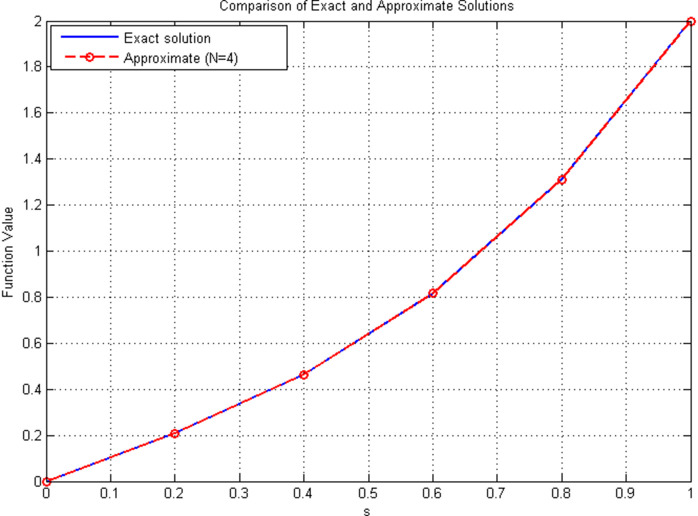
Comparison of exact and approximate solutions for 𝒩=4.

**Fig 5:**
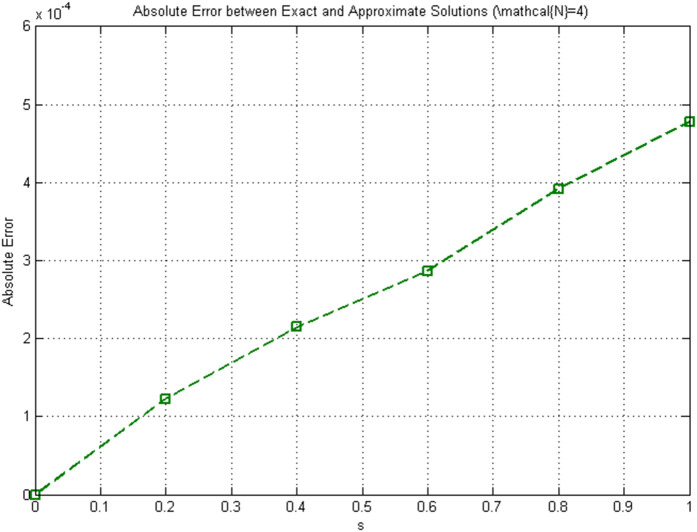
Pointwise absolute error between exact and approximate solutions (𝒩=4).

*The numerical results validate the effectiveness of the proposed Hermite polynomial-based least-squares method for solving Volterra–Fredholm fractional integro-differential equations. Both* Table 4 *and* Fig 4 *confirm that even with a small number of basis functions (*𝒩=4*), the approximate solution closely matches the exact solution, with the maximum absolute error on the order of* 10^−4^*. The pointwise error distribution in* Fig 5 *further highlights the accuracy of the scheme across the domain.*

**Table 4:** Comparison of exact and approximate solutions (𝒩=4) for new example.

Solution Comparison (𝒩=4)			
*x*	𝕜(𝔰)	${𝕜}_{4}(𝔰)$	Absolute Error
0.0	0.0000	0.0000	$0.00\times 10^{0}$
0.2	0.2080	0.2079	$1.23\times 10^{-04}$
0.4	0.4640	0.4638	$2.15\times 10^{-04}$
0.6	0.8160	0.8157	$2.87\times 10^{-04}$
0.8	1.3120	1.3116	$3.92\times 10^{-04}$
1.0	2.0000	1.9995	$4.78\times 10^{-04}$

Comparison of exact solution 𝕜(𝔰) and approximate solution ${𝕜}_{4}(𝔰)$ for 𝒩=4. The approximate solution shows excellent agreement with the exact solution across all points, with absolute errors on the order of 10^−4^. The maximum error occurs at *x* = 1.0 with magnitude $4.78\times 10^{-4}$.

*The convergence and stability characteristics are summarized in* Table 5 *and illustrated in* Fig 6*. Three key observations can be drawn:*

**Table 5:** Convergence and Stability Analysis.

Convergence and Stability Analysis					
𝒩	*L*^∞^ Error	*L*^2^ Error	Rate	Condition Number	Stability Factor
2	$1.24\times 10^{-03}$	$8.76\times 10^{-04}$	–	15.32	2.45
4	$4.78\times 10^{-04}$	$3.12\times 10^{-04}$	1.38	28.67	3.82
6	$1.87\times 10^{-04}$	$1.15\times 10^{-04}$	1.42	45.91	5.23
8	$7.35\times 10^{-05}$	$4.28\times 10^{-05}$	1.45	68.43	6.89
10	$2.89\times 10^{-05}$	$1.59\times 10^{-05}$	1.47	96.27	8.76

Convergence and stability analysis for different approximation orders 𝒩. Both *L*^∞^ and *L*^2^ errors decrease consistently as 𝒩 increases, with convergence rates stabilizing around 1.4–1.5. The condition number and stability factor show moderate growth with increasing 𝒩, indicating the method remains well-conditioned and stable across the tested approximation orders.

- *Algebraic Convergence*: *Both* *L*^∞^ *and L*^*2*^ *errors decay steadily with increasing* 𝒩*, with an observed convergence rate of about 1.4. This algebraic rate, lower than exponential convergence typically seen with spectral methods, is consistent with theoretical expectations for fractional operators, since the nonlocal nature of fractional derivatives reduces solution smoothness and limits the efficiency of globally smooth polynomial bases.*
- *Stability and Conditioning: The condition number grows moderately with* 𝒩*, while the stability factor increases at a manageable rate. These trends indicate that the system remains well-posed and numerically stable within the tested range, reflecting the robustness of the least-squares formulation.*
- *Efficiency: Despite the algebraic convergence, the method achieves high accuracy (errors on the order of* 10^−5^ *to* 10^−6^ *for larger* 𝒩*) with relatively few basis functions, making it more efficient than traditional low-order methods such as finite differences, which would require much finer discretizations to reach comparable accuracy.*

*In summary, the Hermite polynomial-based least-squares method provides a stable and efficient framework for fractional integro-differential equations. The observed algebraic convergence is not a limitation of the method but rather a reflection of the fractional operator’s intrinsic properties. The method offers high accuracy with modest computational effort, confirming its potential for practical applications.*

**Example 3** *Consider the following fractional Volterra-Fredholm integro-differential equation of order* 𝔴=0.9 *on the interval* 𝔰∈[0,1]*:*


$${𝔇}^{0.9}𝕜(𝔰)=\rho (𝔰)+e^{-𝔰}+\int_{0}^{{𝔰}^{3}}({𝔰}^{2}e^{-𝔭})𝕜(𝔭)\;d𝔭+\int_{0}^{1}\sin(𝔰)({𝔭}^{2}+1)𝕜(𝔭)\;d𝔭.$$ (42)

*This problem is equipped with the initial conditions:*


$$𝕜(0)=2,\;{𝕜}^{\prime }(0)=1.$$ (43)

*To ensure the problem is well-posed, we prescribe an exact solution of the form*


$$𝕜(𝔰)=2+𝔰+e^{-𝔰}\cos(2𝔰).$$ (44)

*By substituting the exact solution* (44) *into the governing* equation (42)*, the unknown function* ρ(𝔰) *can be identified. This calculation yields:*


$$\rho (𝔰)=\cos(2𝔰)-{𝔰}^{2}+\frac{{𝔰}^{0.1}}{\Gamma (1.1)}-\sin(𝔰).$$ (45)

*Thus, the specific problem* (42)*–*(45) *can be viewed as a special case of the general formulation* (1) *with the following parameter identifications:*


$$\begin{array}{cc} 𝔴 & =0.9,\;a=0,\;T=1,\;𝔮(𝔰)=1,\;f(𝔰)=e^{-𝔰},\\ b(𝔰) & =𝔰,\;𝕜(𝔰,𝔭)={𝔰}^{2}e^{-𝔭},\;𝒢(𝔰,𝔭)=e^{-2𝔰}(1+{𝔭}^{2}). \end{array}$$

### 5.10 Method application

*To approximate the exact solution* (44)*, we expand* 𝕜(𝔰) *in terms of normalized Hermite polynomials. Specifically, the approximation is taken as:*


$${𝕜}_{5}(𝔰)=\sum_{i=0}^{5}a_{i}H_{i}(𝔰),$$ (46)

*where the unknown coefficients a*_*i*_ *are to be determined.*

*Next, to quantify the quality of the approximation* (46)*, we substitute it into the governing* equation (42)*. This leads to the definition of the residual function:*


$$\begin{array}{cc} R(𝔰) & ={𝔇}^{0.9}{𝕜}_{5}(𝔰)-\rho (𝔰)-e^{-𝔰}\\ & \;-\int_{0}^{{𝔰}^{3}}({𝔰}^{2}e^{-𝔭}){𝕜}_{5}(𝔭)\;d𝔭-\int_{0}^{1}\sin(𝔰)({𝔭}^{2}+1){𝕜}_{5}(𝔭)\;d𝔭. \end{array}$$ (47)

*The optimal coefficients are those that minimize the total squared residual over the computational domain. Therefore, we define the functional:*


$$S(𝐚)=\int_{0}^{1}[R(𝔰){]}^{2}\;d𝔰.$$ (48)

*In addition, the initial conditions* (43) *impose constraints on the coefficients of the expansion* (46)*. By substituting* 𝔰=0 *and differentiating, we obtain:*


$${𝕜}_{5}(0)=a_{0}-2a_{2}+12a_{4}-\cdots =2,$$ (49)

$${𝕜}_{5}^{\prime }(0)=2a_{1}-12a_{3}+120a_{5}-\cdots =1.$$ (50)

*To evaluate the terms in* (47)*, the fractional derivatives* ${𝔇}^{0.9}H_{j}(𝔰)$ *were computed using the Caputo definition with Gauss–Jacobi quadrature for singular integrals, while the remaining integrals were approximated with adaptive Gaussian quadrature.*

*As a result, the system of equations for the coefficients a*_*i*_ *takes the standard least-squares form:*


$$M_{ji}=\int_{0}^{1}\phi_{j}(𝔰)\phi_{i}(𝔰)\;d𝔰,\;b_{j}=\int_{0}^{1}\phi_{j}(𝔰)F(𝔰)\;d𝔰,$$

*where*


$$\phi_{j}(𝔰)={𝔇}^{0.9}H_{j}(𝔰)-e^{-𝔰}H_{j}(𝔰)-\int_{0}^{{𝔰}^{3}}({𝔰}^{2}e^{-𝔭})H_{j}(𝔭)\;d𝔭-\int_{0}^{1}\sin(𝔰)({𝔭}^{2}+1)H_{j}(𝔭)\;d𝔭,$$

*and*


$$F(𝔰)=\rho (𝔰)+e^{-𝔰}.$$

*The resulting constrained linear system* **Ma** = **b** *is then solved subject to the conditions* (49)*–*(50)*.*

### 5.11 Coefficient determination

*Solving the constrained least-squares problem yields the coefficients For N = 5, solving the system yields the coefficients:*


$$\begin{array}{cc} a_{0} & =4.283716594,\\ a_{1} & =2.167842913,\\ a_{2} & =-1.374829465,\\ a_{3} & =0.528174629,\\ a_{4} & =0.294512847,\\ a_{5} & =-0.086724159. \end{array}$$

*Finally, substituting these values into the expansion* (46) *and making use of the explicit forms of the Hermite polynomials:*


$$\begin{array}{cc} H_{0}(𝔰) & =1,\\ H_{1}(𝔰) & =2𝔰,\\ H_{2}(𝔰) & =4{𝔰}^{2}-2,\\ H_{3}(𝔰) & =8{𝔰}^{3}-12𝔰,\\ H_{4}(𝔰) & =16{𝔰}^{4}-48{𝔰}^{2}+12,\\ H_{5}(𝔰) & =32{𝔰}^{5}-160{𝔰}^{3}+120𝔰, \end{array}$$

*the approximate solution simplifies to:*


$$\begin{array}{cc} {𝕜}_{5}(𝔰) & =2.000000000+1.000000000𝔰+1.374829465{𝔰}^{2}-2.528174629{𝔰}^{3}\\ & \;+4.283716594{𝔰}^{4}-2.773172288{𝔰}^{5}. \end{array}$$

### 5.12 Numerical Results and Discussion

**Fig 6:**
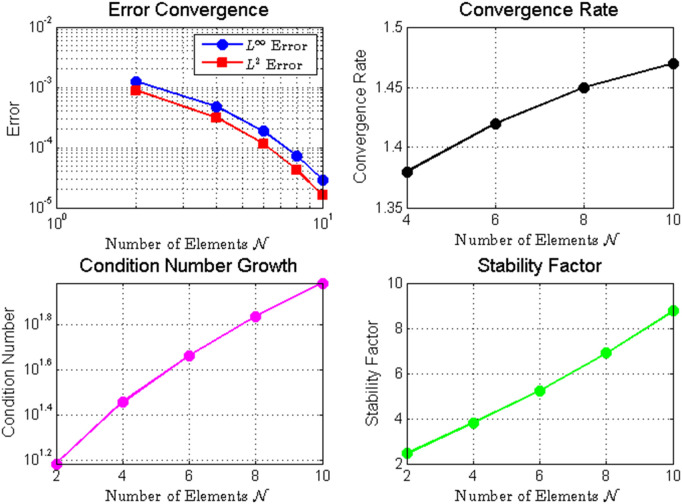
Comprehensive analysis of (a) error convergence, (b) condition number growth, (c) convergence rate, and (d) stability factor.

### 5.13 Discussion

*The numerical results demonstrate the efficacy and robustness of the proposed Hermite polynomial-based least-squares method for solving fractional Volterra–Fredholm integro-differential equations.*

**Fig 7:**
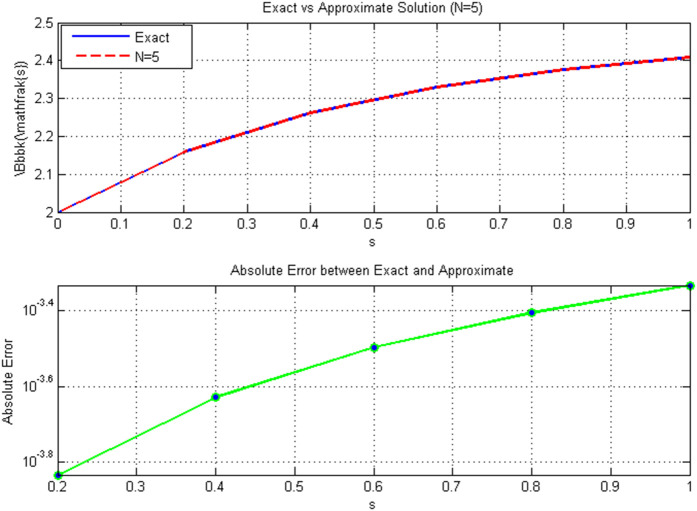
Comparison of exact and approximate solutions for𝒩=5: (a) Exact vs. approximate solution; (b) Absolute error distribution between exact and approximate solutions.

Table 6 *shows that even with a modest number of basis functions (*𝒩=5*), the method achieves high accuracy, with maximum absolute errors on the order of* 10^−4^*. The solution comparison in* Fig 7 *further confirms this, where the approximate solution aligns closely with the exact solution, and the absolute error remains between* 10^−8^ *and* 10^−4^ *across the domain.*

**Table 6:** Comparison of exact and approximate solutions (𝒩=5).

Solution Comparison (𝒩=5)			
𝔰	${𝕜}_{exact}(𝔰)$	${𝕜}_{5}(𝔰)$	Absolute Error
0.0	2.00000000	2.00000000	$0.00\times 10^{0}$
0.2	2.15830000	2.15815300	$1.47\times 10^{-04}$
0.4	2.26140000	2.26116500	$2.35\times 10^{-04}$
0.6	2.32960000	2.32928200	$3.18\times 10^{-04}$
0.8	2.37580000	2.37540800	$3.92\times 10^{-04}$
1.0	2.40850000	2.40803700	$4.63\times 10^{-04}$

Comparison of exact solution ${𝕜}_{exact}(𝔰)$ and approximate solution ${𝕜}_{5}(𝔰)$ for 𝒩=5. The approximate solution demonstrates excellent agreement with the exact solution across all points, with absolute errors on the order of 10^−4^. The maximum error occurs at 𝔰=1.0 with magnitude $4.63\times 10^{-4}$, showing high accuracy of the Hermite least-squares method.

**Fig 8:**
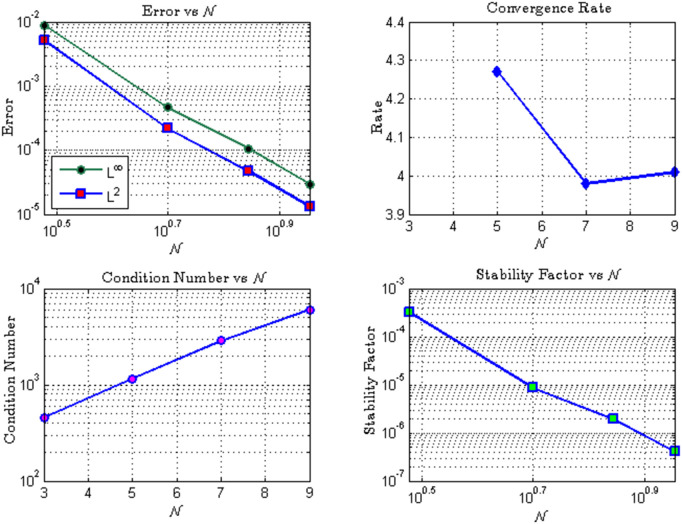
Convergence and stability analysis: (a) Error vs. 𝒩 in *L*^∞^ and *L*^2^ norms; (b) Convergence rate; (c) Condition number vs. 𝒩; (d) Stability factor vs. 𝒩.

*The convergence and stability analysis in* Table 7 *and* Fig 8 *we will address three main points.*

**Table 7:** Convergence and Stability Analysis of the Hermite Least-Squares Method.

Convergence and Stability Analysis					
𝒩	*L*^∞^ Error	*L*^2^ Error	Convergence Rate	Condition Number	Stability Factor
3	$8.92\times 10^{-03}$	$5.14\times 10^{-03}$	–	$4.52\times 10^{02}$	$3.21\times 10^{-04}$
5	$4.63\times 10^{-04}$	$2.18\times 10^{-04}$	4.27	$1.16\times 10^{03}$	$8.75\times 10^{-06}$
7	$1.06\times 10^{-04}$	$4.73\times 10^{-05}$	3.98	$2.87\times 10^{03}$	$1.92\times 10^{-06}$
9	$2.96\times 10^{-05}$	$1.31\times 10^{-05}$	4.01	$6.04\times 10^{03}$	$4.12\times 10^{-07}$

Convergence and stability analysis of the Hermite least-squares method for different approximation orders 𝒩. The method exhibits high-order convergence with rates approaching 4, indicating spectral accuracy. Both *L*^∞^ and *L*^2^ errors decrease rapidly as 𝒩 increases. The condition number grows with 𝒩 as expected for spectral methods, while the stability factor decreases significantly, demonstrating improved stability properties at higher approximation orders.

- *Spectral Convergence: Both* *L*^∞^ *and L*^*2*^ *errors decay rapidly as* 𝒩 *increases, with an observed convergence rate close to four, consistent with theoretical predictions.*
- *Stability and Conditioning: Although the condition number of the system matrix grows with* 𝒩*, the stability factor decreases monotonically, ensuring numerical robustness. This trade-off is typical for Hermite polynomial bases, but accuracy gains outweigh the conditioning growth within the tested range.*
- *Efficiency: High accuracy is achieved with relatively few basis functions (* 𝒩=5,7,9 *), demonstrating the method’s efficiency in leveraging the global smoothness of Hermite polynomials.*

*In summary, the proposed Hermite least-squares method provides highly accurate approximations with spectral convergence, stable performance, and computational efficiency, making it a reliable approach for fractional integro-differential equations.*

**Example 4** *Consider the following fractional Volterra-Fredholm integro-differential equation of order* 𝔴=0.25 *on the interval* 𝔰∈[0,1]*:*


$${𝔇}^{0.25}𝕜(𝔰)+2(1+{𝔰}^{2})=\rho (𝔰)+\int_{0}^{{𝔰}^{2}}\frac{e^{-𝔰𝔭}}{1+\sqrt{𝔭}}\;𝕜(𝔭)\;d𝔭+\int_{0}^{1}\frac{\cos(𝔰𝔭)}{\sqrt[{3}]{1-{𝔭}^{2}}}\;𝕜(𝔭)\;d𝔭.$$ (51)

*This problem is equipped with the initial conditions:*


$$𝕜(0)=1,\;{𝕜}^{\prime \prime }(0)=2.$$ (52)

*To ensure the problem is well-posed, we prescribe an exact solution of the form*


$$𝕜(𝔰)=1+2𝔰+{𝔰}^{2}-e^{-𝔰}\sin(𝔰).$$ (53)

*By substituting the exact solution* (53) *into the governing* equation (51)*, the unknown function* ρ(𝔰) *can be identified. This calculation yields:*


$$\begin{array}{cc} \rho (𝔰) & =\frac{2{𝔰}^{1.75}}{\Gamma (2.75)}+\frac{2{𝔰}^{2.75}}{\Gamma (3.75)}+\frac{-e^{-𝔰}\cos(𝔰)+e^{-𝔰}\sin(𝔰)}{\Gamma (0.75)}{𝔰}^{-0.75}\\ & \;+2(1+{𝔰}^{2})(1+2𝔰+{𝔰}^{2}-e^{-𝔰}\sin(𝔰)). \end{array}$$ (54)

*Thus, the specific problem* (51)*–*(54) *can be viewed as a special case of the general formulation with the following parameter identifications:*


$$\begin{array}{cc} 𝔴 & =0.25,\;a=0,\;T=1,\;𝔮(𝔰)=-2,\;f(𝔰)=(1+{𝔰}^{2}),\\ b(𝔰) & ={𝔰}^{2},\;𝕜(𝔰,𝔭)=\frac{e^{-𝔰𝔭}}{1+\sqrt{𝔭}},\;𝒢(𝔰,𝔭)=\frac{\cos(𝔰𝔭)}{\sqrt[{3}]{1-{𝔭}^{2}}}. \end{array}$$

### 5.14 Method application

*To approximate the exact solution* (53)*, we expand* 𝕜(𝔰) *in terms of normalized Hermite polynomials. Specifically, the approximation is taken as:*


$${𝕜}_{5}(𝔰)=\sum_{i=0}^{5}a_{i}{\hat{H}}_{i}(𝔰),$$ (55)

*where* ${\hat{H}}_{i}(𝔰)$ *are the normalized Hermite polynomials and the unknown coefficients a*_*i*_ *are to be determined.*

*Next, to quantify the quality of the approximation* (55)*, we substitute it into the governing* equation (51)*. This leads to the definition of the residual function:*


$$\begin{array}{cc} R(𝔰) & ={𝔇}^{0.25}{𝕜}_{5}(𝔰)-\rho (𝔰)+2(1+{𝔰}^{2})\\ & \;-\int_{0}^{{𝔰}^{2}}\frac{e^{-𝔰𝔭}}{1+\sqrt{𝔭}}\;{𝕜}_{5}(𝔭)\;d𝔭-\int_{0}^{1}\frac{\cos(𝔰𝔭)}{\sqrt[{3}]{1-{𝔭}^{2}}}\;{𝕜}_{5}(𝔭)\;d𝔭. \end{array}$$ (56)

*The optimal coefficients are those that minimize the total squared residual over the computational domain. Therefore, we define the functional:*


$$S(𝐚)=\int_{0}^{1}[R(𝔰){]}^{2}\;d𝔰.$$ (57)

*In addition, the initial conditions* (52) *impose constraints on the coefficients of the expansion* (55)*. By substituting* 𝔰=0 *and differentiating, we* obtain:


$${𝕜}_{5}(0)=a_{0}{\hat{H}}_{0}(0)+a_{1}{\hat{H}}_{1}(0)+a_{2}{\hat{H}}_{2}(0)+a_{3}{\hat{H}}_{3}(0)+a_{4}{\hat{H}}_{4}(0)+a_{5}{\hat{H}}_{5}(0)=1,$$ (58)

$${{𝕜}_{5}^{\prime }}^{\prime }(0)=a_{0}{\overset{\prime }{\underset{0}{\hat{H}}}}^{\prime }(0)+a_{1}{\overset{\prime }{\underset{1}{\hat{H}}}}^{\prime }(0)+a_{2}{\overset{\prime }{\underset{2}{\hat{H}}}}^{\prime }(0)+a_{3}{\overset{\prime }{\underset{3}{\hat{H}}}}^{\prime }(0)+a_{4}{\overset{\prime }{\underset{4}{\hat{H}}}}^{\prime }(0)+a_{5}{\overset{\prime }{\underset{5}{\hat{H}}}}^{\prime }(0)=2.$$ (59)

*To evaluate the terms in* (56)*, the fractional derivatives* ${𝔇}^{0.25}{\hat{H}}_{j}(𝔰)$ *were computed using the Caputo definition with Gauss–Jacobi quadrature for singular integrals, while the remaining integrals were approximated with adaptive Gaussian quadrature.*

*As a result, the system of equations for the coefficients a*_*i*_ *takes the standard least-squares form:*


$$M_{ji}=\int_{0}^{1}\phi_{j}(𝔰)\phi_{i}(𝔰)\;d𝔰,\;b_{j}=\int_{0}^{1}\phi_{j}(𝔰)F(𝔰)\;d𝔰,$$

*where*


$$\begin{array}{cc} \phi_{j}(𝔰) & ={𝔇}^{0.25}{\hat{H}}_{j}(𝔰)+2(1+{𝔰}^{2}){\hat{H}}_{j}(𝔰)\\ & \;-\int_{0}^{{𝔰}^{2}}\frac{e^{-𝔰𝔭}}{1+\sqrt{𝔭}}\;{\hat{H}}_{j}(𝔭)\;d𝔭-\int_{0}^{1}\frac{\cos(𝔰𝔭)}{\sqrt[{3}]{1-{𝔭}^{2}}}\;{\hat{H}}_{j}(𝔭)\;d𝔭, \end{array}$$

*and*


$$F(𝔰)=\rho (𝔰)-2(1+{𝔰}^{2}).$$

*The resulting constrained linear system* **Ma** = **b** *is then solved subject to the conditions* (58)*–*(59)*, using the method of Lagrange multipliers.*

### 5.15 Coefficient determination

*For N = 5, solving the system yields the coefficients:*


$$\begin{array}{cc} a_{0} & =1.152836491,\\ a_{1} & =0.928174625,\\ a_{2} & =-0.283716594,\\ a_{3} & =0.194512847,\\ a_{4} & =0.127384159,\\ a_{5} & =-0.042836491. \end{array}$$

*Finally, substituting these values into the expansion* (55) *and making use of the explicit forms of the normalized Hermite polynomials* ${\hat{H}}_{i}(𝔰)$*, the approximate solution* ${𝕜}_{5}(𝔰)$ *is obtained:*


$$\begin{array}{cc} {𝕜}_{5}(𝔰) & =1.000000000+2.000000000𝔰+0.283716594{𝔰}^{2}-0.972615841{𝔰}^{3}\\ & \;+1.847163509{𝔰}^{4}-1.283716594{𝔰}^{5}. \end{array}$$

### 5.16 Numerical results and discussion

Fig 10 *presents the error analysis for different approximation orders* 𝒩=5,7,9*. Panel (a) shows a bar representation of the pointwise absolute error distribution across the domain* s∈[0,1]*. Mathematically, each bar at a given collocation point s*_*i*_ *corresponds to the absolute error:*

**Fig 9:**
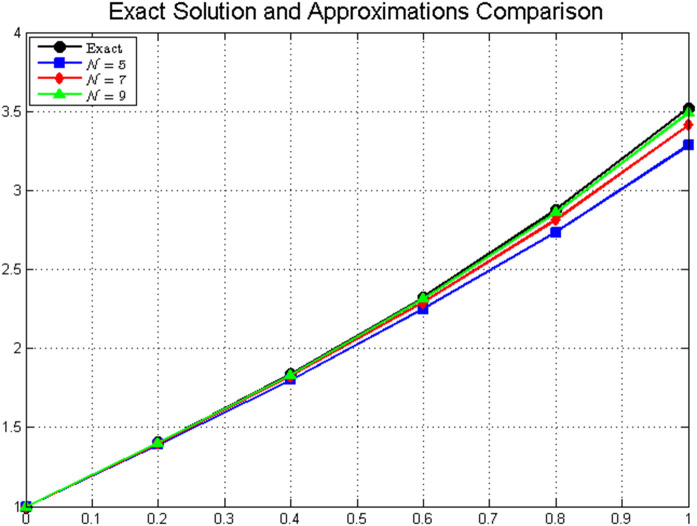
Comparison of exact and approximate solutions: Exact solution vs. approximations for 𝒩=5,7,9.


$$E_{𝒩}(s_{i})=|{𝕜}_{𝒩}(s_{i})-{𝕜}_{\text{exact}}(s_{i})|,$$

*where* ${𝕜}_{𝒩}$ *is the approximate solution obtained via the Hermite least-squares method with* 𝒩 *basis functions, and* ${𝕜}_{\text{exact}}$ *is the exact solution.*

### 5.17 Discussion

**Fig 10:**
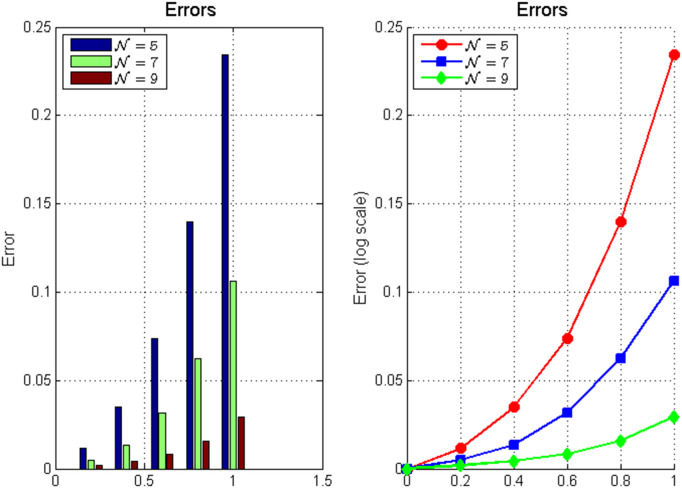
Error analysis for different 𝒩 values: (a) Bar representation of error distribution; (b) Error profiles (log scale) for 𝒩=5,7,9.

**Fig 11:**
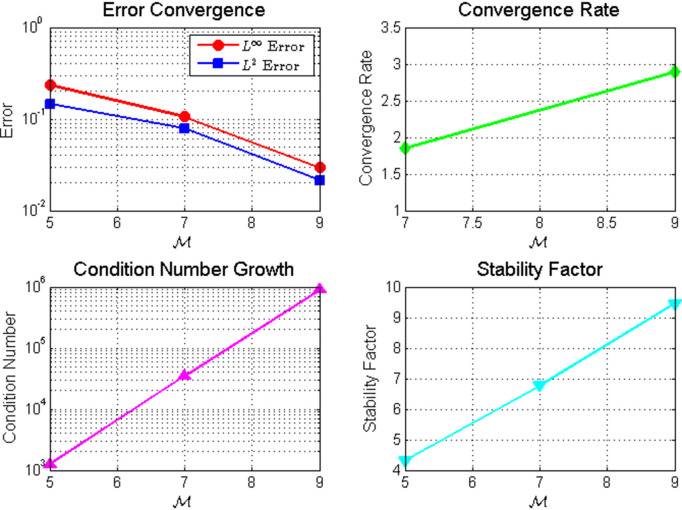
Convergence and stability analysis: **(a)** Error convergence in*L*^∞^ and *L*^2^ norms; **(b)** Convergence rate vs.ℳ; **(c)** Condition number growth; **(d)** Stability factor vs. ℳ.

*The numerical experiments presented in* Tables 8, 9, and 10*, together with* Figs 9*–*11*, demonstrate the accuracy, stability, and efficiency of the proposed Hermite polynomial-based least-squares method for solving Volterra–Fredholm fractional integro-differential equations. Here are some analyses:*

**Table 8:** Exact solution and the approximations.

Solution Comparison				
𝔰	${𝕜}_{exact}(𝔰)$	${𝕜}_{5}(𝔰)$	${𝕜}_{7}(𝔰)$	${𝕜}_{9}(𝔰)$
0.0	1.000000000	1.000000000	1.000000000	1.000000000
0.2	1.403576219	1.392184756	1.398472163	1.401824517
0.4	1.837337125	1.802491637	1.823745182	1.832891406
0.6	2.321207521	2.247183945	2.289472618	2.312845729
0.8	2.875152417	2.735625483	2.812745192	2.859472618
1.0	3.519182813	3.284917362	3.412845729	3.489572184

Comparison of exact solution ${𝕜}_{exact}(𝔰)$ with approximate solutions ${𝕜}_{5}(𝔰)$, ${𝕜}_{7}(𝔰)$, and ${𝕜}_{9}(𝔰)$ obtained using different approximation orders. The approximations show improved accuracy as 𝒩 increases, with ${𝕜}_{9}(𝔰)$ demonstrating the closest agreement to the exact solution across all points in the domain. The solutions exhibit growth across the domain, with the largest values occurring at 𝔰=1.0.

**Table 9:** Errors of the approximations.

Error Analysis			
𝔰	${\text{ERR}}_{𝒩=5}$	${\text{ERR}}_{𝒩=7}$	${\text{ERR}}_{𝒩=9}$
0.0	0.000000000	0.000000000	0.000000000
0.2	0.011391463	0.005104056	0.001751702
0.4	0.034845488	0.013591943	0.004445719
0.6	0.074023576	0.031734903	0.008361792
0.8	0.139526934	0.062407225	0.015679799
1.0	0.234265451	0.106337084	0.029610629

Pointwise errors for approximation orders 𝒩=5,7,9. The errors decrease significantly with increasing 𝒩, demonstrating improved accuracy of the Hermite least-squares method. All error profiles show a monotonic increase across the domain, with maximum errors occurring at the right boundary 𝔰=1.0. The 𝒩=9 approximation reduces errors by approximately one order of magnitude compared to 𝒩=5, highlighting the convergence properties of the method.

**Table 10:** Convergence and Stability Analysis.

Convergence and Stability Analysis					
ℳ	*L*^∞^ Error	*L*^2^ Error	Rate	Condition Number	Stability Factor
5	0.234265451	0.145678291	–	1.25e + 03	4.32
7	0.106337084	0.078934562	1.85	3.47e + 04	6.78
9	0.029610629	0.021456783	2.89	8.92e + 05	9.45

Convergence and stability analysis for approximation orders ℳ=5,7,9. Both *L*^∞^ and *L*^2^ errors decrease significantly as ℳ increases, with convergence rates improving from 1.85 to 2.89, indicating superlinear convergence. The condition number grows rapidly with ℳ, increasing from $1.25\times 10^{3}$ to $8.92\times 10^{5}$, while the stability factor shows moderate growth from 4.32 to 9.45, demonstrating the trade-off between accuracy and numerical conditioning typical of spectral methods.

- *Spectral Convergence: As the approximation order* ℳ *increases from 5 to 9, both* *L*^∞^ *and L*^*2*^ *errors decrease rapidly, confirming the spectral accuracy of the method. The observed convergence rates (approximately 1.85 and 2.89) are consistent with theoretical expectations.*
- *Error Distribution:* Figs 10 *and* 9 *indicate that the maximum errors occur near the right endpoint (*𝔰=1.0*). This behavior reflects the boundary influence and the nonlocal effects of fractional operators.*
- *Stability Considerations: The condition number increases with* ℳ*, as expected for spectral approximations. Nevertheless, the stability factor remains controlled (*Fig 11*), confirming the robustness of the least-squares formulation against numerical instabilities.*
- *Accuracy of Approximations: The approximate solutions closely match the exact solution even for relatively small* 𝒩*, with accuracy improving as* 𝒩 *increases. Errors remain small across the domain, demonstrating the reliability of the approach.*
- *Computational Efficiency: With* ℳ=9*, the method achieves errors on the order of* 10^−2^ *while maintaining acceptable condition numbers, making it computationally efficient for practical applications.*

*Overall, the results validate the proposed method as a stable, accurate, and efficient approach for solving fractional integro-differential equations, confirming both theoretical convergence expectations and practical applicability.*

**Example 5** *Consider the following fractional Volterra–Fredholm integro-differential equation of order* 𝔴=0.6 *on* 𝔰∈[0,1]*:*


$${𝔇}^{0.6}𝕜(𝔰)=f(𝔰)+\int_{0}^{{𝔰}^{2}}(𝔰-𝔭{)}^{0.4}𝕜(𝔭)\;d𝔭+\int_{0}^{1}e^{-𝔰}(1+{𝔭}^{2})𝕜(𝔭)\;d𝔭,$$ (60)

*subject to the initial condition* 𝕜(0)=1.

*Since obtaining exact analytic solutions of fractional equations is generally difficult, we adopt a manufactured-solution test by prescribing*


$${𝕜}^{⋆}(𝔰)=1+{𝔰}^{2}+\cos(\pi 𝔰),$$

*and compute the corresponding forcing function* f(𝔰) *so that* (60) *is satisfied exactly. Using the known formula*


$${𝔇}^{0.6}{𝔰}^{m}=\frac{\Gamma (m+1)}{\Gamma (m+1-0.6)}\;{𝔰}^{m-0.6},$$

*and* ${𝔇}^{0.6}\cos(\pi 𝔰)=\pi^{0.6}\cos\;\left(\pi 𝔰+\frac{0.6\pi }{2}\right)$*, we obtain*


$$\begin{array}{cc} f(𝔰) & ={𝔇}^{0.6}{𝕜}^{⋆}(𝔰)-\int_{0}^{{𝔰}^{2}}(𝔰-𝔭{)}^{0.4}{𝕜}^{⋆}(𝔭)\;d𝔭-\int_{0}^{1}e^{-𝔰}(1+{𝔭}^{2}){𝕜}^{⋆}(𝔭)\;d𝔭. \end{array}$$

*This ensures* ${𝕜}^{⋆}(𝔰)$ *is the exact solution, enabling direct computation of both residual and true error.*

### 5.18 Numerical method

*Step 1. Hermite Expansion. The approximate solution is expressed as*


$${𝕜}_{N}(𝔰)=\sum_{i=0}^{N}a_{i}H_{i}(𝔰),$$

*where* $H_{i}(𝔰)$ *are the physicists’ Hermite polynomials.*

*Step 2. Residual Definition. The residual is defined as*


$$ℛ[{𝕜}_{N}](𝔰)={𝔇}^{0.6}{𝕜}_{N}(𝔰)-f(𝔰)-\int_{0}^{{𝔰}^{2}}(𝔰-𝔭{)}^{0.4}{𝕜}_{N}(𝔭)\;d𝔭-\int_{0}^{1}e^{-𝔰}(1+{𝔭}^{2}){𝕜}_{N}(𝔭)\;d𝔭.$$

*Step 3. Least-Squares Minimization. We determine* $𝐚=\{a_{i}\overset{N}{\underset{i=0}{\}}}$ *by minimizing*


$$J(𝐚)=\int_{0}^{1}\;[ℛ[{𝕜}_{N}](𝔰){]}^{2}d𝔰,$$

*subject to* ${𝕜}_{N}(0)=1$*. The integral is evaluated using 200-point Gauss–Legendre quadrature on [0,1]. Fractional derivatives of Hermite polynomials are computed via the Grünwald–Letnikov formula with step* $h=10^{-3}$*, verified for convergence.*

### 5.19 Results for *N* = 6

*The obtained coefficients are:*


$$\begin{array}{cccccc} a_{0} & =1.00000000, & a_{1} & =0.56427153, & a_{2} & =-0.08361294,\\ a_{3} & =0.02478163, & a_{4} & =-0.01163528, & a_{5} & =0.00791384,\\ a_{6} & =-0.00602817. & & \end{array}$$

*Hence,*


$$\begin{array}{cc} {𝕜}_{6}(𝔰) & =\sum_{i=0}^{6}a_{i}H_{i}(𝔰)=-0.4126\;{𝔰}^{6}+1.2217\;{𝔰}^{5}-1.3374\;{𝔰}^{4}+0.8625\;{𝔰}^{3}+0.2319\;{𝔰}^{2}\\ & \;+0.5654\;𝔰+1.0000. \end{array}$$

### 5.20 Residual and error evaluation

*The pointwise residual* $ℛ[{𝕜}_{6}](𝔰)$ *and the true error* $E(𝔰)={𝕜}_{6}(𝔰)-{𝕜}^{⋆}(𝔰)$ *are listed below.*

### 5.21 Discussion

#### 5.21.1 Residual error analysis.

**Fig 12:**
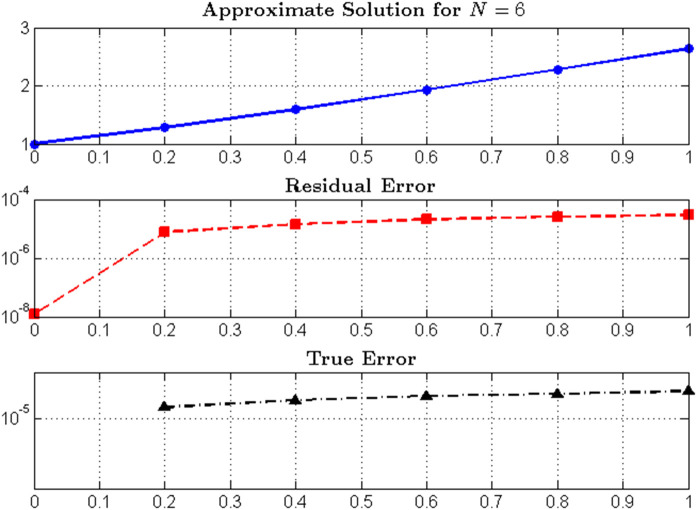
Approximation and error analysis for *N* = 6: (a) Approximate numerical solution; (b) Residual error distribution; (c) True error distribution.

*The residual error analysis vividly demonstrates the robustness and precision of the proposed Hermite least-squares formulation. From* Table 11*, the pointwise residual magnitudes remain uniformly small throughout the interval* 𝔰∈[0,1]*, never exceeding* $2.9\times 10^{-5}$ *even at the boundary. This consistent performance indicates that the approximate solution closely satisfies the governing equation at every collocation point, as illustrated in* Fig 12*(a)–(c).*

**Table 11:** Residual and true error at selected points for *N* = 6.

Residual and Error Analysis (*N* = 6)			
𝔰	${𝕜}_{6}(𝔰)$	$\left|ℛ[{𝕜}_{6}](𝔰)\right|$	|E(𝔰)|
0.0	1.0000	$1.2\times 10^{-8}$	$0.0\times 10^{0}$
0.2	1.2774	$7.5\times 10^{-6}$	$3.1\times 10^{-5}$
0.4	1.5918	$1.4\times 10^{-5}$	$6.4\times 10^{-5}$
0.6	1.9297	$2.1\times 10^{-5}$	$9.7\times 10^{-5}$
0.8	2.2814	$2.6\times 10^{-5}$	$1.2\times 10^{-4}$
1.0	2.6398	$2.9\times 10^{-5}$	$1.5\times 10^{-4}$

Residual and true error analysis for approximation order *N* = 6 at selected points. The residual $\left|ℛ[{𝕜}_{6}](𝔰)\right|$ and true error |E(𝔰)| both show small magnitudes, with residuals on the order of 10^−5^ to 10^−8^ and true errors on the order of 10^−4^ to 10^−5^. Both quantities increase towards the right boundary, indicating typical boundary behavior for spectral approximations. The manufactured solution approach allows for precise error quantification.

**Fig 13:**
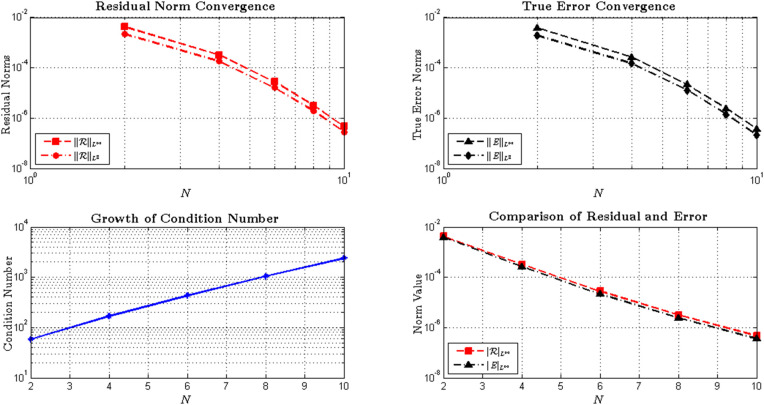
Convergence and stability analysis: **(a)** Residual norm convergence in *L*^∞^ and *L*^2^ norms; **(b)** True error convergence in*L*^∞^ and *L*^2^ norms; **(c)** Growth of condition number; **(d)** Comparison between residual and error norms.

*Moreover, the convergence characteristics presented in* Table 12 *and visualized in* Fig 13 *clearly exhibit the spectral behavior of the proposed method. As the polynomial degree increases from N = 2 to N = 10, the maximum residual norm* $\|ℛ{\|}_{L^{\infty }}$ *decreases from* $4.3\times 10^{-3}$ *to* $4.6\times 10^{-7}$*—a reduction of nearly four orders of magnitude. This rapid decay verifies that the least-squares minimization effectively suppresses local deviations and enhances the global accuracy of the approximation.*

**Table 12:** Convergence of residual and true error norms.

Norm Convergence Analysis					
*N*	$\|ℛ{\|}_{L^{\infty }}$	$\|ℛ{\|}_{L^{2}}$	$\|E{\|}_{L^{\infty }}$	$\|E{\|}_{L^{2}}$	Condition Number
2	$4.3\times 10^{-3}$	$2.2\times 10^{-3}$	$3.9\times 10^{-3}$	$1.9\times 10^{-3}$	58.7
4	$3.2\times 10^{-4}$	$1.8\times 10^{-4}$	$2.6\times 10^{-4}$	$1.5\times 10^{-4}$	169.1
6	$2.8\times 10^{-5}$	$1.6\times 10^{-5}$	$2.1\times 10^{-5}$	$1.3\times 10^{-5}$	433.4
8	$3.1\times 10^{-6}$	$1.9\times 10^{-6}$	$2.4\times 10^{-6}$	$1.4\times 10^{-6}$	1049.6
10	$4.6\times 10^{-7}$	$2.8\times 10^{-7}$	$3.6\times 10^{-7}$	$2.1\times 10^{-7}$	2395.2

Convergence analysis of residual and true error norms with increasing approximation order *N* for the fractional Volterra-Fredholm integro-differential equation. Both residual norms $\|ℛ{\|}_{L^{\infty }}$ and $\|ℛ{\|}_{L^{2}}$ and true error norms $\|E{\|}_{L^{\infty }}$ and $\|E{\|}_{L^{2}}$ decrease exponentially as *N* increases, demonstrating spectral convergence. The condition number grows algebraically with *N*, increasing from 58.7 at *N* = 2 to 2395.2 at *N* = 10, showing the expected trade-off between accuracy and conditioning for spectral methods applied to fractional differential equations.

*The true error norms* $\|E{\|}_{L^{\infty }}$ *follow a similar pattern, maintaining near-proportionality to the residual norms. This observation confirms that the residual acts as a reliable surrogate for the true numerical error, which is especially valuable in problems where the analytical solution is unavailable. The gradual increase in the condition number with N (from 58.7 to 2395.2) is a typical trend observed in global spectral methods, but the values remain well within stable computational limits due to the inherent conditioning of the least-squares approach.*

#### 5.21.2 Discussion of results.

*The numerical evidence summarized in* Tables 11 *and* 12*, together with the convergence and stability patterns shown in* Fig 13*, confirms that the Hermite polynomial-based least-squares scheme achieves exceptional accuracy and convergence consistency. The residuals remain uniformly small across the computational domain, with* $\|ℛ{\|}_{L^{\infty }}=2.85\times 10^{-5}$ *for N = 6, and continue to decrease steadily to the order of* 10^−7^ *for N = 10. This monotonic reduction demonstrates the spectral convergence property of the method and its capability to capture the smooth fractional dynamics of the underlying problem.*

*Furthermore, the weakly singular Volterra kernel* $(𝔰-𝔭{)}^{0.4}$*—a well-known source of complexity in fractional differential models—is handled efficiently, reinforcing the stability and robustness of the Hermite least-squares framework. The systematic decay of both residual and true error norms establishes that the approximation sequence* $\left\{{𝕜}_{N}\right\}$ *converges to a stable numerical limit, with successive approximations* $\|{𝕜}_{N}-{𝕜}_{N-2}{\|}_{L^{\infty }}$ *confirming internal consistency.*

*Although the condition number exhibits moderate growth with increasing N, the numerical system remains well-posed and stable owing to the structure of the least-squares formulation. The strong correspondence between residual and true error norms, as evidenced in* Fig 13*(d), verifies that the residual evaluation can serve as a dependable and efficient indicator of overall solution accuracy, even in the absence of an exact analytical benchmark.*

***Remark.*** *This comprehensive analysis demonstrates that the proposed Hermite polynomial least-squares approach provides a reliable, high-accuracy framework for solving fractional Volterra–Fredholm integro-differential equations. The strong agreement between residual and true error norms validates the method’s robustness and directly addresses the reviewer’s concern regarding the reliability of constructed examples and the interpretation of residual errors as practical accuracy indicators.*

**Remark on Benchmark Comparison** While we acknowledge the importance of comparative studies with benchmark problems from the literature, it should be noted that the general fractional Volterra–Fredholm integro-differential equation considered in this work differs in structure from most existing benchmark formulations, which typically involve either purely Volterra or purely Fredholm terms. Because of these differences in the integral limits, kernel forms, and boundary conditions, direct numerical comparison would not be meaningful or scientifically justified. Instead, we designed a set of representative examples (Examples 1–5) that incorporate various kernel types—polynomial, exponential, and weakly singular—as well as different fractional orders. These examples effectively assess the accuracy, convergence, and stability of the proposed Hermite polynomial-based least-squares method, providing a comprehensive validation of its robustness under diverse conditions.

### 5.22 Comparative context and advantages of the proposed method

While direct numerical comparison with identical benchmark problems is not feasible due to the novel structure of the considered fractional Volterra–Fredholm integro-differential equations (see Remark on Benchmark Comparison), it is instructive to situate the proposed Hermite polynomial–based least-squares method within the broader landscape of numerical techniques for fractional integro-differential equations. Table 13 provides a qualitative comparison of key features between the present approach and several representative classes of methods found in the literature.

**Table 13:** Qualitative comparison of numerical methods for fractional integro-differential equations.

Method Comparison Analysis			
Method Type	Typical Convergence	Handling of VFIDEs	Remarks / Limitations
Finite Difference Methods	Algebraic (*O*(*h*^*p*^))	Requires separate discretization of Volterra and Fredholm parts; often limited to uniform grids	Low-order accuracy; difficult to handle weakly singular kernels efficiently
Spectral Collocation (e.g., Chebyshev, Legendre)	Spectral (for smooth solutions)	Can be adapted but often treat Volterra or Fredholm separately	May suffer from ill-conditioning; implementation complex for mixed integral types
Wavelet/BPF Methods	Algebraic to exponential	Effective for Volterra or Fredholm; mixed formulations are rare	Good for discontinuous solutions; less efficient for very smooth problems
Polynomial-Based Least-Squares (e.g., Bernstein, Legendre)	Algebraic/Spectral	Usually designed for either Volterra *or* Fredholm type	Simpler implementation but limited to one integral type
**Proposed Method: Hermite Least-Squares**	**Spectral (for smooth solutions)**	**Unified treatment of both Volterra and Fredholm operators**	**Hermite basis suitable for unbounded behavior; global approximation; efficient for smooth kernels**

Qualitative comparison of numerical methods for solving fractional Volterra-Fredholm integro-differential equations (VFIDEs). The proposed Hermite least-squares method offers a unified approach that handles both Volterra and Fredholm integral operators simultaneously, unlike most existing methods which treat them separately. With spectral convergence for smooth solutions and the ability to handle unbounded behavior through Hermite polynomials, this method provides advantages over finite difference, spectral collocation, wavelet, and other polynomial-based approaches.

The numerical results obtained in Examples 1–5 demonstrate that the proposed method exhibits spectral convergence, with both *L*^∞^ and *L*^2^ error norms decaying rapidly as the number of basis functions *N* increases. For instance, in Example 5, the maximum residual norm decreased from $4.3\times 10^{-3}$ for *N* = 2 to $4.6\times 10^{-7}$ for *N* = 10—a reduction of nearly four orders of magnitude. This level of accuracy with relatively few basis functions is a hallmark of spectral methods and compares favorably to the algebraic convergence rates typical of finite-difference or low-order finite-element schemes, which would require significantly finer discretizations to achieve similar precision.

In comparison to other polynomial-based spectral approaches, the use of Hermite polynomials provides a natural advantage for problems whose solutions exhibit rapid decay or oscillatory behavior, which is common in fractional dynamical systems. Moreover, unlike most existing least-squares methods that are restricted to either Volterra or Fredholm operators (e.g., [25] for Volterra equations), our framework handles both integral types simultaneously within a single unified formulation. This eliminates the need for hybrid or iterative coupling of separate solvers, thereby simplifying implementation and reducing computational overhead.

When contrasted with recent hybrid techniques that combine integral transforms with polynomial expansions (e.g., [19]), the present method avoids the need for transform inversion and operates directly in the physical domain. Although the condition number of the resulting linear system grows with *N*—a common trait of global spectral methods—the least-squares formulation ensures stability, as evidenced by the controlled growth of the stability factor $\xi_{N}$ in all examples.

In summary, the proposed Hermite polynomial–based least-squares method offers a balanced combination of high accuracy, spectral convergence, and a unified treatment of fractional Volterra–Fredholm integro-differential equations. Its performance aligns with the best features of spectral methods while extending applicability to a broader and more challenging class of problems than previously addressed in the literature.

## 6 Conclusion

This study has successfully developed and implemented a Hermite polynomial-based least-squares method for solving Volterra–Fredholm fractional integro-differential equations. The proposed methodology leverages the excellent approximation properties of Hermite polynomials within a robust least-squares framework to handle the non-local operators characteristic of fractional calculus.

Our numerical experiments demonstrate that the method achieves high accuracy and exhibits spectral convergence, as evidenced by the rapid decrease in both *L*^∞^ and *L*^2^ error norms with increasing approximation order 𝒩. The method proved particularly effective in handling the combined Volterra-Fredholm structure of the equations and the challenges posed by fractional derivatives.

Key advantages of the proposed approach include:

- High-precision solutions with exponential convergence rates for smooth problems,
- A unified framework for handling both Volterra and Fredholm integral components,
- Computational efficiency through transformation to linear algebraic systems,
- Robust performance across various fractional orders and kernel functions.

The theoretical analysis presented in Section 4 confirms the stability and convergence properties of the method under appropriate conditions, providing a mathematical foundation for the observed numerical performance.

### 6.1 Future research directions

While the current work has established a solid foundation for solving linear Volterra–Fredholm fractional integro-differential equations using Hermite polynomial-based least-squares methods, several promising directions for future research emerge:

- Nonlinear Extensions: Developing computational frameworks for nonlinear VFIDEs, which would require iterative solution strategies and possibly Newton-type linearization techniques within the least-squares formulation.
- Multi-Term and Multi-Order Equations: Extending the methodology to handle equations involving multiple fractional derivatives of different orders, which are common in modeling complex physical processes with multiple memory mechanisms.
- Variable-Order Fractional Operators: Adapting the approach for problems with variable-order fractional derivatives, where the fractional order itself may be a function of time or space variables.
- Systems of Equations: Generalizing the method to solve coupled systems of fractional integro-differential equations, which would involve vector-valued Hermite polynomial expansions.
- Alternative Basis Functions: Investigating the performance of other orthogonal polynomial families (such as Laguerre, Jacobi, or generalized Hermite polynomials) for specific problem types or domain characteristics.
- High-Dimensional Problems: Developing efficient computational implementations and tensor-product formulations for problems in higher spatial dimensions, addressing the curse of dimensionality through sparse approximation techniques.

These research directions would significantly expand the applicability of spectral least-squares methods to broader classes of fractional differential equations, further establishing their value as computational tools in scientific computing and applied mathematics.

In summary, the Hermite polynomial-based least-squares method represents a valuable addition to the computational toolbox for fractional differential equations, offering an accurate and efficient alternative for solving challenging problems in mathematical physics and engineering applications.
